# Extracellular Vesicles in Musculoskeletal Pathologies and Regeneration

**DOI:** 10.3389/fbioe.2020.624096

**Published:** 2021-01-20

**Authors:** Marietta Herrmann, Solvig Diederichs, Svitlana Melnik, Jana Riegger, Drenka Trivanović, Shushan Li, Zsuzsa Jenei-Lanzl, Rolf E. Brenner, Markus Huber-Lang, Frank Zaucke, Frank A. Schildberg, Susanne Grässel

**Affiliations:** ^1^Interdisciplinary Center for Clinical Research (IZKF) Group Tissue Regeneration in Musculoskeletal Diseases, University Hospital Würzburg, Würzburg, Germany; ^2^Bernhard-Heine-Centrum for Locomotion Research, University of Würzburg, Würzburg, Germany; ^3^Research Centre for Experimental Orthopaedics, Centre for Orthopaedics, Trauma Surgery and Paraplegiology, Heidelberg University Hospital, Heidelberg, Germany; ^4^Division for Biochemistry of Joint and Connective Tissue Diseases, Department of Orthopedics, University of Ulm, Ulm, Germany; ^5^Department of Orthopedic Surgery, Experimental Orthopedics, Centre for Medical Biotechnology (ZMB), University of Regensburg, Regensburg, Germany; ^6^Dr. Rolf M. Schwiete Research Unit for Osteoarthritis, Orthopedic University Hospital Friedrichsheim, Frankfurt, Germany; ^7^Institute of Clinical and Experimental Trauma-Immunology, University Hospital of Ulm, Ulm, Germany; ^8^Clinic for Orthopedics and Trauma Surgery, University Hospital Bonn, Bonn, Germany

**Keywords:** extracellular vesicles, exosomes, musculoskeletal diseases, MSC, iMP, cell-free therapeutics

## Abstract

The incidence of musculoskeletal diseases is steadily increasing with aging of the population. In the past years, extracellular vesicles (EVs) have gained attention in musculoskeletal research. EVs have been associated with various musculoskeletal pathologies as well as suggested as treatment option. EVs play a pivotal role in communication between cells and their environment. Thereby, the EV cargo is highly dependent on their cellular origin. In this review, we summarize putative mechanisms by which EVs can contribute to musculoskeletal tissue homeostasis, regeneration and disease, in particular matrix remodeling and mineralization, pro-angiogenic effects and immunomodulatory activities. Mesenchymal stromal cells (MSCs) present the most frequently used cell source for EV generation for musculoskeletal applications, and herein we discuss how the MSC phenotype can influence the cargo and thus the regenerative potential of EVs. Induced pluripotent stem cell-derived mesenchymal progenitor cells (iMPs) may overcome current limitations of MSCs, and iMP-derived EVs are discussed as an alternative strategy. In the last part of the article, we focus on therapeutic applications of EVs and discuss both practical considerations for EV production and the current state of EV-based therapies.

## Introduction

**Figure 1:**
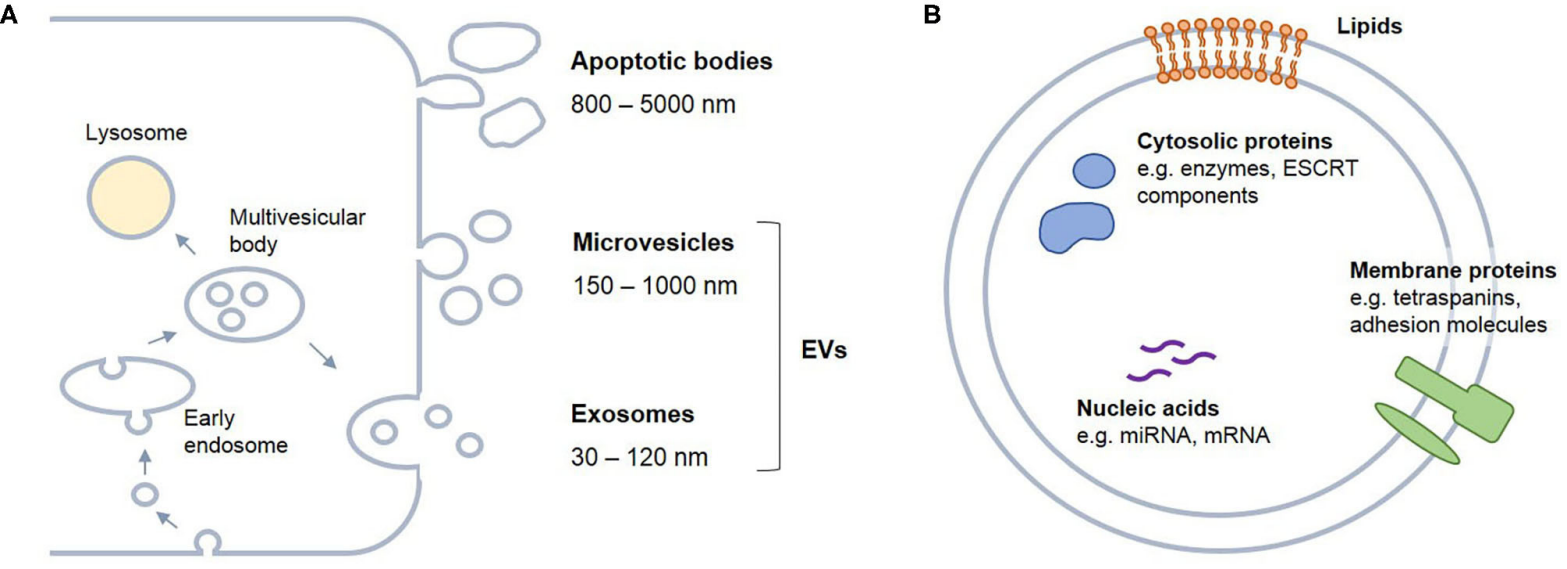
Biogenesis and content of extracellular vesicles (EVs). EVs are secreted by any cell type. **(A)** Based on the biogenesis route, different kinds of vesicles can be distinguished. Apoptotic bodies arise from membrane blebbing of apoptotic cells. Microvesicles derive from outward budding of the plasma membrane, while exosomes originate from the endo-lysosomal compartment and are released from the cell after fusion of multivesicular bodies with the plasma membrane. **(B)** EVs are enclosed by a lipid bilayer and carry membrane lipids and proteins, cytosolic proteins as well as nucleic acids, such as miRNAs and mRNAs. It is widely assumed that EV cargo mirrors the characteristics of the parent cell. ESCRT, endosomal sorting complexes required for transport.

In the past years, the musculoskeletal research field has seen an increased interest in extracellular vesicles (EVs), due to their prognostic and therapeutic potential. The term EVs describes vesicles of a broad size range from 30 to 5,000 nm in diameter enclosed by a lipid bilayer. Different types of vesicles are distinguished based on their biogenesis route (Figure 1A). Apoptotic bodies, sized 800–5,000 nm, arise from membrane blebbing and fragmentation of apoptotic cells (Xu et al., 2019b). Microvesicles (MVs), also referred to as ectosomes, microparticles, or shedding vesicles, range between 150 and 1,000 nm in diameter and are formed by outward budding of the cell membrane (Colombo et al., 2014). The most homogenous vesicle type are exosomes, which originate from the endolysosomal compartment. Invagination of vesicles into multivesicular bodies (MVBs) and their release after fusion with the plasma membrane gives rise to exosomes with a diameter between 30 and 120 nm (Thery et al., 2002). Due to overlapping size ranges and limited specificity of current enrichment and detection methods, in most cases MVs and exosomes cannot be distinguished and both are referred to as small EVs.

EV secretion has been shown for virtually any cell type. EVs carry proteins, lipids and nucleic acids (Figure 1B) and thus are critically involved in cell-to-cell communication. EVs participate in autocrine, paracrine and systemic signaling processes and have accordingly been detected in most body fluids. Mechanisms by which EVs can elicit cellular responses are highly variable and may depend on EV type, cargo, as well as their cellular origin. Direct release of cargo into the extracellular space has been described, as well as adhesion and/or uptake by target cells by micropinocytosis or clathrin-mediated endocytosis in a receptor-dependent or -independent manner (Cocucci and Meldolesi, 2015; McKelvey et al., 2015). After uptake, the vesicles might enter the lysosomal pathway or fuse with endocytic membranes resulting in the release of their cargo into the cytoplasm (Prada and Meldolesi, 2016).

There is increasing evidence for a critical role of EVs in progression of musculoskeletal diseases as well as tissue regeneration. Here, we aim to summarize the role of EVs in musculoskeletal tissues. Mesenchymal stromal cells (MSCs) have been in the focus of therapeutic strategies for musculoskeletal diseases for many years. Accordingly, next to potential mechanisms and pathways targeted by EVs, we discuss MSCs as a cell source of EVs and particularly address the parameters, which might affect the phenotype of MSC-derived EVs (MSC-EVs). We further provide practical considerations for developing EVs as a cell-free, therapeutic tool and review examples of therapeutic applications in musculoskeletal diseases.

## Role of EVs In Musculoskeletal Regeneration—From Pro-Regenerative to Detrimental Effects

### Matrix Remodeling and Mineralization

EVs are generated by almost any cell type and can contain a large variety of different cargoes. Databases like, e.g., Vesiclepedia (http://microvesicles.org) and ExoCarta (http://www.exocarta.org) provide regularly updated lists of proteins, lipids, and nucleic acids that have been identified in EVs from different sources. The exact composition of EVs derived from a specific cell type depends on the microenvironment in the tissue that might undergo changes during development or due to pathological conditions (Cheng et al., 2017; Kusuma et al., 2017).

With regard to remodeling and mineralization, EVs can contribute in several fundamentally different ways: (1) EVs themselves transport remodeling or mineralization factors, or carry the remodeling activities on their surface. (2) The activities of EV membrane-associated proteinases and other enzymes generate cleavage products or mobilize growth factors that activate cells. (3) EVs stimulate cells via membrane receptors or phagocytosis in an auto- or paracrine manner to release factors that modulate remodeling and mineralization.

Several studies have indicated the presence of different matrix metalloproteinases (MMPs) in and on EVs (Shimoda and Khokha, 2013, 2017). With regard to matrix remodeling, MMP-2,−9,−13, and−14 could be of particular importance as MMP-2 and−9 are gelatinases and all aforementioned MMPs share collagenolytic activity. MMP-14, also referred to as MT1-MMP, is a transmembrane protein that acts as a sheddase by cleaving other transmembrane molecules relevant in cell-cell and cell-matrix interactions, such as the intercellular adhesion molecule 1 (ICAM-1, aka CD54), the hyaluronan receptor (CD44), and the growth factor-binding heparan-sulfate proteoglycan syndecan-1 (CD138) (Shimoda and Khokha, 2017). MMPs are often activated in cascades, and a recent study has shown that exosomal MMP-14 is able to cleave and activate pro-MMP-2 (Hakulinen et al., 2008). Furthermore, tissue inhibitor of metalloproteinases 3 (TIMP3)-sensitive aggrecanase activity has been found in vesicles derived from rheumatoid synovial fibroblasts, and it has been speculated that this aggrecanase activity might play an important role in remodeling aggrecan-rich matrices (Lo Cicero et al., 2012). The activity of MMPs might not only degrade cell surface molecules but also result in the release of bioactive cleavage fragments that can further modulate remodeling processes (Karamanos et al., 2019). In addition to proteases, glycosidases like sialidases, and heparanases have been detected on the exosomal surface (Sumida et al., 2015; Bandari et al., 2018; Sanderson et al., 2019). These enzymes degrade a variety of glycosaminoglycans and glycoproteins in the extracellular space where they might not only contribute to matrix degradation but also to the concomitant release of growth factors that are often bound to glycan structures.

The crosslinking of collagens and elastin by lysyl oxidases is a relevant process in stabilizing extracellular matrices (ECM) in various musculoskeletal tissues. Indeed, a member of the lysyl oxidase family, lysyl-oxidase-like 2 (LOXL2), has been detected on EVs derived from endothelial cells. Interestingly, under hypoxic conditions an increased enzymatic LOXL2 activity was detected (de Jong et al., 2016), demonstrating how an altered microenvironment could affect matrix stability. In rheumatoid arthritis (RA) and osteoarthritis (OA), not only the decreased oxygen concentration but also pro-inflammatory cytokines in the joint might similarly contribute to EV-mediated remodeling processes (Fernandes et al., 2002; Ng et al., 2010; Goldring and Otero, 2011). Treatment of chondrocytes with interleukin-1β (IL-1β) *in vitro*, mimicking an inflammatory OA environment, induces a release of EVs that stimulates synovial fibroblasts to produce 3-fold higher levels of MMP-13 in comparison to untreated control cells (Kato et al., 2014). This observation confirms the role of EVs as messengers in intercellular communication (Raposo and Stoorvogel, 2013).

In bone, all different cell types have been shown to be involved in the remodeling process via EVs. In addition to bone synthesis and resorption, EVs actively participate in the regulation of ECM mineralization (Masaoutis and Theocharis, 2019). During endochondral ossification, EVs play an important role in the initiation of the mineralization in the growth plate. These vesicles were first described by Anderson (1967, 1969) and Bonucci (1969) in the late 1960s and are still referred to as matrix vesicles. Calcium, phosphate and phospholipids form a nucleation core complex in the lumen of EVs derived from chondrocytes and hydroxyapatite crystals start to grow. The exact molecular mechanism of mineralization is still not known, but eventually collagen fibrils in the ECM become mineralized (Azoidis et al., 2018; Rilla et al., 2019). Since EVs can even contribute to bone formation in adult rats (Hoshi and Ozawa, 2000), many investigations are currently focusing on therapeutic applications using EVs in fracture repair (see section EV Therapy in Fracture Healing).

### Angiogenic Effects of EVs

Angiogenesis, defined as the formation of new vessels by sprouting and ingrowth from the existing vascular network, is critical for the regeneration of vascularized tissues, with bone being one prominent example. Angiogenesis involves several processes, including proliferation, recruitment, and migration of endothelial cells as well as stabilization of newly formed vessels by mural cells, such as pericytes. Effects of EVs have been described for all of these steps. EVs with a role in angiogenesis may derive from endothelial cells and their progenitors, platelets, various immune cell populations, and MSCs. They have been reported to exert pro- or antiangiogenic effects (Todorova et al., 2017) that have been related to various signaling pathways, which may be targeted by the corresponding ligands and cytokines contained in EVs, such as: vascular endothelial growth factor (VEGF) (Brill et al., 2005; Zou et al., 2016; Gangadaran et al., 2017, 2020; Ahn et al., 2018; Tang et al., 2019), platelet-derived growth factor (PDGF) (Brill et al., 2005; Ma et al., 2017) and MMPs (Taraboletti et al., 2002; Han et al., 2019; Tang et al., 2019). Moreover, a number of miRNAs involved in angiogenic effects of EVs have been identified, e.g., pro-angiogenic miR31, miR-125a, miR-126, miR-130a, miR-150, miR-210, miR214 (Li et al., 2013; van Balkom et al., 2013; Kang et al., 2016; Liang et al., 2016; Lombardo et al., 2016; Du et al., 2017a; Gong et al., 2017a; Wu et al., 2018; Jia et al., 2019; Nie et al., 2019; Gangadaran et al., 2020; Zhu et al., 2020).

Abundance of VEGF and subsequent targeting of the VEGF receptor pathway is one of the mechanisms, which have been accounted for the pro-angiogenic effects of EVs (Gangadaran et al., 2017). Also, Notch pathway is well-known to be involved in angiogenesis. Delta-like (Dll) 1, 3, and 4 and Jagged 1 and 2 are membrane bound ligands that bind to the Notch receptor resulting in cleavage of its intracellular domain and subsequent transcriptional changes (Luo et al., 2019). Different Notch-ligands and regulators thereof have been identified in EVs and associated with pro- (Liang et al., 2016; Gonzalez-King et al., 2017) or anti-angiogenic effects (Tan et al., 2018). More signaling pathways have been connected to the pro-angiogenic activity of EVs, including Erk1/2 (Brill et al., 2005; Shabbir et al., 2015; Lombardo et al., 2016; Zhang et al., 2016b; Wu et al., 2018; Jia et al., 2019; Tang et al., 2019), PKA signaling (Xue et al., 2018), PI3K/Akt signaling (Shabbir et al., 2015; Liu et al., 2017a; Ma et al., 2017; Ji et al., 2019), Wnt4/βCatenin (Zhang et al., 2015a) and NFκB (Anderson et al., 2016; Nie et al., 2019).

As also discussed in the section MSC as a Source of EVs—Influence of “MSC State” on EVs, the microenvironment of parental cells of EVs may have a significant impact on EV composition. This knowledge can be used to optimize EV-based therapeutic approaches, for example, with respect to promoting their angiogenic effects. Next to pro-angiogenic impact induced by hypoxic preconditioning (Xue et al., 2018; Han et al., 2019; Zhu et al., 2020), it has been demonstrated that nitric oxide (NO) stimulation could augment the pro-angiogenic effects of placenta-derived MSCs, which was attributed to increased levels of miR-126 and VEGF (Du et al., 2017a). Similarly, PDGF stimulation promotes the pro-angiogenic efficacy of adipose tissue MSC-EVs (Lopatina et al., 2014).

### Immunomodulatory Effects of EVs

EVs are important players in immune regulation and thereby play a key role in musculoskeletal pathologies and regeneration. Depending on the origin and state of the EV-releasing cell (Robbins and Morelli, 2014; Burrello et al., 2016), they contribute to both activation and suppression of immune responses. EVs, especially originating from bone marrow MSCs (BMSCs), have been heavily studied because of the immunomodulatory capacity of MSCs and the hopes that MSC-EVs could be used as a potent cell-free immune modulatory agent without facing the hurdles of cell transplantation. Indeed, several *in vitro* and *in vivo* studies could prove the immunosuppressive effects of EVs, and how they interfere with innate, as well as adaptive, immune cells (Siegel et al., 2009; Gomzikova et al., 2019). EVs can modulate the functionality of T cells, B cells, dendritic cells (DC), macrophages, natural killer cells (NK), and others (Burrello et al., 2016). Interestingly, some of the therapeutic effects mediated by EVs are comparable to their cell of origin (Fierabracci et al., 2015), which suggests that EVs carry a similar molecular composition and that the immune modulatory effect can be transferred (Di Trapani et al., 2016). Recent research could show that EVs contain tolerogenic molecules, such as programmed death-ligand 1 (PD-L1), galectin-1, and transforming growth factor (TGF)-β1 (Mokarizadeh et al., 2012). However, it should not be concealed that EVs have been described to also activate immune responses, for example, by transferring antigens and the major histocompatibility complex (MHC) (Andre et al., 2004).

EVs modulate immune cell function in different ways: They can inhibit the proliferation of CD4^+^ and CD8^+^ T cells and the release of interferon (IFN)-γ and tumor necrosis factor (TNF)-α (van den Akker et al., 2018). Furthermore, EVs can alter T helper (Th) cell differentiation by fostering the differentiation of Th2 out of Th1 cells and by inhibiting Th17 cell differentiation (Blazquez et al., 2014; Chen et al., 2016; Ji et al., 2019). Lastly, EVs have been shown to induce regulatory T cell generation, which can further regulate ongoing immune responses (Zhang et al., 2014, 2018a; Ji et al., 2019). This phenomenon is dependent on TGF-β and can be blocked by neutralizing antibodies (Alvarez et al., 2018).

The extent of EV-mediated immune regulation seems to be relative to the amount of received EVs: the more EVs are incorporated by the acceptor cell, the more phenotypic changes could be seen (Di Trapani et al., 2016). B cells have been described to be an exceptionally good recipient for EVs. As reaction to EV uptake, B cells showed reduced proliferation and differentiation. Of further note, the proliferation of NK cells can be reduced by EVs (Di Trapani et al., 2016), demonstrating their immunomodulatory potential not only for adaptive immune cells, but also in the innate immune system.

Recent investigations indicated that EVs affect the antigen presenting cell (APC) properties of DCs. In addition to compromising antigen uptake, they also diminished the maturation of DCs, which subsequently resulted in a reduced immune response. In line with this, EVs decreased the production of pro-inflammatory cytokines and increased the release of the anti-inflammatory cytokine TGF-β (Reis et al., 2018). Similarly, EVs also modulate macrophage function by inducing an anti-inflammatory M2-like phenotype (Henao Agudelo et al., 2017). It has been shown that preconditioning the EV-releasing cell can enhance the ability of EVs to influence macrophage differentiation. Examples are hypoxia and LPS challenge which increased the amount of MSC-EV-associated miR-223 and miR-146b, as well as let-7b (Ti et al., 2015; Lo Sicco et al., 2017), respectively, thereby leading to M2 polarization. Of note, these effects appear to be age-dependent as discussed later (see section The Impact of Senescence and Age-Related Changes). However, several other mechanisms have been published describing how EVs can tweak macrophage biology not only by involving single effector molecules, but even mitochondrial delivery (Morrison et al., 2017). Although MSC-EVs exert a variety of immune inhibitory properties, EVs also stimulate the innate immune system, such as by inducing M1 macrophage polarization during infection (Brauer et al., 2020).

Because of their potential to negatively and positively interfere with innate and adaptive immune responses, it is no surprise that EVs, on the one hand, actively contribute to pathologies in the musculoskeletal system and, on the other hand, are currently under consideration to be exploited as therapeutic approaches in regenerative medicine.

EVs have not only great therapeutic but also high prognostic potential. Current research showed that knowledge about the abundance and content of EVs could be used to improve our understanding of several inflammatory diseases, especially in situations where the immune system destroys tissue integrity, such as in RA (Knijff-Dutmer et al., 2002; Skriner et al., 2006; Rodriguez-Carrio et al., 2015; Maeda et al., 2017; Murphy et al., 2018). Another example where EVs may serve as a biomarker, are musculoskeletal infections. A recent study demonstrated that synovial fluid of periprosthetic joints contains EVs, and that they differ in number, size and composition in the presence of an infection, as well as might influence the local immune microenvironment (Ruwald et al., 2020).

In summary, EVs have a profound effect on a variety of immune processes and thereby contribute to health and disease in the musculoskeletal apparatus.

### Senescence-Associated Vesicle Release

In response to stress signals, cells can restore their functions, or undergo a cell death program to prevent persistence of damaged and transformed cells (Takahashi et al., 2017; Gorgoulis et al., 2019). On the other side, the cellular senescence program enables survival of cells with permanent changes affecting their microenvironment. Appearance of the senescence-associated secretory phenotype (SASP) is followed by the activation of a distinct transcriptional landscape in comparison to primary senescence (Kirschner et al., 2020) and secretion of high levels of inflammatory cytokines, chemokines, and MMPs (Salama et al., 2014) contributing to various age-related pathologies. Although distinct cell lineages within the bone microenvironment (osteoblasts, B cells, T cells) can dramatically increase SASP-associated molecule production, particularly myeloid cells and osteocytes of aged mice, contribute to age-related bone loss (Farr et al., 2016), regulating osteoblast mineralization and osteoclastogenesis (Farr et al., 2017).

In addition to the secretion of inflammatory molecules, the release of EVs is also upregulated in senescent cells. A recent study demonstrated that senescence-associated upregulation of EV biogenesis, along with autophagy, can block apoptosis and thus has a critical impact on cell homeostasis (Hitomi et al., 2020). RNA-sequencing and liquid chromatography-mass (LCM) spectrometry analysis revealed that several SASP proteins, including TGF-β2, osteoprotegrin (OPG), MMP-9, TIMP1, macrophage migration inhibitory factor (MIF)-1, peroxiredoxins (PRDXs), and insulin-like growth factor binding protein (IGFBP) 3, were upregulated in EVs derived from aged mouse osteocytes (Zhang et al., 2019; Aquino-Martinez et al., 2020). A proteomic analysis of the senescence-associated secretome of fibroblasts further revealed a significant abundance of plasma membrane proteins in EVs (Basisty et al., 2020). Concomitantly, an increase of all cargo miRNAs, which predominantly target mRNAs of pro-apoptotic proteins, might be related to the anti-apoptotic activity of senescent cell-derived EVs. In addition, senescence-specific differences in miRNA composition of fibroblast EVs was observed, where miRNAs were identified which were selectively retained in senescent cells (miR-21-3p and miR-17-3p), or packaged into senescent cell-derived EVs (miR-15b-5p and miR-30a-3p) (Terlecki-Zaniewicz et al., 2018; Alibhai et al., 2020).

Aging can significantly alter the miRNA cargo of EVs in the bone marrow microenvironment, which may, in turn, play a role in stem cell senescence and osteogenic differentiation. Thus, comparison of EVs derived from BMSCs isolated from young (2–4 months) or aged (24–28 months) mice showed enrichment of a miR-96/-182/-183 cluster in aged BMSC-EVs (Davis et al., 2017). EVs derived from osteocytes isolated from 3 and 20 months old mice, revealed similar distribution of “exosomal proteins,” including tetraspanins, flotillin, caveolin, integrins, annexins, transcription factors (EF1A and EF2), heat shock proteins and phosphatidylserine-binding protein, while “aged” osteocyte-EVs lacked proteins involved in the maintenance of cell homeostasis, regulation of cellular metabolism and osteoblast as well as osteoclast differentiation. Furthermore, EVs from “aged” osteocytes contained several factors, including CD44, CD47, CD59, TGF-β2, and gelsolin, which regulate the activities of both immune cells and bone cells, implying important communication between “aged” osteocytes and immune cells (Zhang et al., 2019).

Aged skeletal muscle is a potential source of circulating, senescence-associated EVs expressing CD63 and TSG101 proteins that may impact on stem cell populations in bone via miR-34a *in vivo* and *ex vivo*. It has been shown that senescent muscle-derived EVs can induce cellular senescence in BMSCs, revealing a potential mode of inter-organ crosstalk that may affect bone physiology with aging (Fulzele et al., 2019). Together, these findings suggest that EVs isolated from aged cells possess certain specificities, and thus, consideration of donor age as additional criteria for EVs' isolation is desirable.

### EVs in Bone Healing and Homeostasis

The native process of bone healing proceeds through overlapping stages of inflammation, repair, and remodeling, which involve multiple signaling pathways acting in concert within the bone defect and the surrounding soft tissues (Oryan et al., 2017). Immigrating MSCs into the bone defect secrete large amounts of EVs and it was shown that EV-based approaches have the potential to promote bone regeneration (see section EV Therapy in Fracture Healing). These pleiotropic, mostly anabolic effects, modulate metabolism and activity of all bone cell types during osteogenic differentiation resulting in changes of bone turnover. With respect to osteogenesis, the effects of MSC-EVs were inhibited in the presence of LY294002 (phosphoinositide 3-kinase inhibitor), implicating the involvement of PI3K/AKT signaling in EV-induced osteogenesis (Zhang et al., 2016c). In addition, mitogen-activated protein kinase (MAPK), BMP/Smad, and Wnt/β-catenin signaling pathways are involved in regulating EV-induced osteogenesis (Tan et al., 2020). These effects may be at least partly attributed to the protein cargo of MSC-EVs, which accounts for more than 850 different proteins, including TGF-β, insulin-like growth factor (IGF), PDGF and growth differentiation factor (GDF) (Lai et al., 2012; Toh et al., 2017). Numerous *in vitro* studies reported positive effects of MSC-EVs on cell survival including anti-apoptotic effects (Tan et al., 2020), proliferation and/or migration of a variety of cell types as osteoblasts, macrophages, MSCs, and endothelial cells.

Important for exerting anabolic bone remodeling effects is the role of EV cargo in the mutual communication of bone cells, where the components of the EVs differ according to the parental cell type. It was demonstrated that BMSC-derived PKH67-labeled EVs were internalized by osteoblasts and got distributed in diverse organelles promoting bone regeneration (Qin et al., 2016). The other way round, EVs derived from mineralized osteoblasts promote osteoblastic differentiation of BMSCs by activating the Wnt signaling pathway (Cui et al., 2016). Osteocytes secrete EVs containing miRNAs that circulate in the blood and may function as signaling molecules. Several miRNAs, such as miR-29, miR-484, and miR-221, exist in EVs secreted by mouse osteocyte-like cells (MLO-Y4 cells) (Sato et al., 2017). EVs produced by osteocytic cells, which were preincubated with myostatin could be taken up by osteoblastic MC3T3 cells. This internalization led to a clear reduction of Runx2 expression, the key regulator of osteoblastic differentiation, and subsequently, to decreased osteoblastic differentiation via down-regulation of the Wnt signaling pathway. Notably, the inhibitory effect of myostatin-modified osteocytic EVs on osteoblast differentiation was reversed by expression of exogenous miR-218 through a mechanism involving miR-218-mediated inhibition of sclerostin (SOST) gene expression (Qin et al., 2017). As discussed before (see section Angiogenic Effects of EVs), EVs support angiogenesis at different levels, these pro-angiogenic effects have likewise been proofed beneficial in the context of bone regeneration (Xu et al., 2019a; Liu et al., 2020). In this line, local ischemia is the main pathological incident in osteonecrosis of the femoral head (ONFH). There is currently no effective therapy to promote angiogenesis in the femoral head. When iPS-MSC-exosomes were i.v. injected to a steroid-induced rat osteonecrosis model they significantly prevented bone loss, and increased microvessel density in the femoral head compared with the control group (Liu et al., 2017a). Additionally, iPS-MSC-EVs significantly enhanced the proliferation, migration and tube-forming capacities of endothelial cells *in vitro*. The authors conclude that the angiogenesis promoting effect might be attributed to activation of the PI3K/Akt signaling pathway in endothelial cells.

Taken together, MSC-EVs are carriers for biomolecules which transfer messages to other cells in the bone microenvironment and modulate bone homeostasis. These EVs exert potent effects on cell survival, proliferation, and migration, to either promote or delay bone regeneration.

### EVs in Osteoporosis

Osteoporosis is a systemic multifactorial skeletal disorder that results in fragile bones and an increased risk of fragility fractures with minimal trauma. The pathogenesis is complex and includes environmental and genetic factors. Osteoporotic fractures are due to decreased bone mineral density (BMD) in combination with deteriorated bone microarchitecture. Usually, BMD remains stable until the age of about 55 years after which age-related bone loss starts. Bone remodeling is a continuous physiological process whereby old bone is removed by osteoclasts, and new bone is formed by osteoblasts. This process is strictly regulated by numerous signaling pathways (Almeida, 2012; Hendrickx et al., 2015).

Several studies demonstrated that miRNAs released from EVs may contribute to osteoporosis and age-related bone loss indicating that it depends on the cell source whether EVs might rather stimulate bone formation, or bone degradation. Notably, EVs secreted by bone degrading cells, i.e., osteoclasts, are able to suppress osteoblast activity by miRNAs. Elderly women with fractures and ovariectomized mice (*in vivo* model for post-menopausal bone loss) have elevated levels of EVs derived from osteoclasts transporting miR-214-3p (Li et al., 2016). Presumably, inhibition of miR-214-3p can decrease bone loss during aging and thus delay bone mass reduction resulting in osteoporosis. MiR-183 increases in EVs of BMSCs after exposure to oxidative stress (Davis et al., 2017). These “stressed” EVs can reduce proliferation and osteogenic differentiation of young BMSCs by targeting *Hmox1* (gene encoding for heme oxygenase 1). However, EV proteins are also involved in bone turnover and can deregulate the delicate balance between bone degradation and formation. For example, RANKL can be encapsulated into EVs by human osteoblast cell lines. When treated with parathyroid hormone, osteoblasts secrete more RANKL containing EVs, which stimulate osteoclast differentiation (Deng et al., 2015). In parallel, the number of RANK-containing EVs increased in the late stage of osteoclast differentiation and consequently inhibited osteoclast differentiation *via* RANK-RANKL interactions (Huynh et al., 2016). These examples demonstrate that crosstalk among bone cells is important for bone homeostasis in both physiological and pathophysiological conditions, and is –in part- regulated by EVs and their cargo. Hence, EVs from pathologically altered cells in bone might influence the outcome of treatments by counteracting effects from “healthy” MSC-EVs.

In order to understand the underlying mechanisms of MSC-EV effects, the molecular cargo of EVs was examined regarding its potential to improve osteogenesis, cell survival, and angiogenesis of bone cells and their precursors. It became evident that miRNAs are critically involved in EV mediated effects. Kuang et al. (2019) observed therapeutic efficacy of human umbilical cord (UC) MSC-EVs in osteonecrosis and attributed the anti-apoptotic and pro-survival effects of MSC-EVs to miR-21-mediated PTEN (phosphatase and tensin homolog) downregulation and AKT (protein kinase B) phosphorylation. Yang et al. (2020) reported improved bone histological and structural parameters in a rat model of disuse osteoporosis. This was attributed to increased expression of yes-associated protein (YAP) and inhibition of the Hippo signaling pathway. These effects of the UC-MSC-EVs were partly recapitulated with miR-1263 mimics and abrogated with a miR-1263 inhibitor.

Besides, EV cargo might function as independent diagnostic and prognostic biomarker for bone metabolism. In particular, this was observed for changes in serum concentrations of several EV-miRNAs. The serum concentration of EV-derived miR-214-3p, which prevents osteogenesis and bone formation, is increased in elderly patients with fractures (Li et al., 2016). In osteoporotic patients and elderly individuals, levels of miR-31 are also elevated in serum. It is reported that EV-derived miR-31 is taken up by MSCs and inhibits osteogenic differentiation by decreasing the levels of frizzled-3 (Weilner et al., 2016). Furthermore, it was shown that miR-21-5p serum level was reduced in post-menopausal women with low bone mass (Yavropoulou et al., 2017).

However, large scale prospective clinical studies are needed to find out whether EV-derived miRNAs indeed can serve as biomarkers for bone pathologies.

### EVs in Trauma

In general, acute tissue trauma is characterized by inflammation, cell death, and oxidative stress. Moreover, traumatic injuries lead to early cellular senescence and activation of the innate immune system, in particular the complement system, which potentiates trauma effects (Huber-Lang et al., 2018; Jeon et al., 2019; Riegger et al., 2020). While immunomodulatory and senescence-associated effects of EVs have been discussed in the sections before, the following will address cell death, oxidative stress and complement activation.

Trauma-induced cell death compromises direct necrosis and programmed cell death, such as apoptosis and necroptosis (Zhao et al., 2015; Riegger and Brenner, 2019; Stolberg-Stolberg et al., 2020). Apoptotic cells were found to secrete apoptotic exosome-like vesicles (AEVs), expressing typical markers such as CD63, lysosomal-associated membrane protein 1 (LAMP1) and stress-associated heat shock proteins 70 (HSP70) (Park et al., 2018). These AEVs loaded with “find me” and “eat me” signals, attracting phagocytes and promoting phagocytotic uptake (Caruso and Poon, 2018). Moreover, AEVs might act as vehicles for damage associated molecular patterns (DAMPs) (e.g., DNA and HMGB1), inducing inflammatory response in macrophages (Caruso and Poon, 2018; Park et al., 2018). Besides AEVs, various studies reported about EVs released from necroptotic cells—also referred to as “necroptotic bodies” (Zargarian et al., 2017). As these EVs predominantly carry the executive effector protein of the necroptotic pathway, mixed lineage kinase domain-like protein (MLKL), researchers suggest that the EVs result from the shedding of MLKL-affected plasma membranes, which might serve as a survival mechanism (Gong et al., 2017b; Yoon et al., 2017). Comparable to AEVs, uptake of these “necroptotic bodies” by macrophages or APCs results in inflammatory signaling, chemotaxis, and antigen presentation to T cells (Shlomovitz et al., 2020). Taken together, both apoptotic and necroptotic cell-derived EVs might play an important role in immunomodulation and clearance of dysfunctional or dying cells (Zargarian et al., 2017; Caruso and Poon, 2018).

Concerning oxidative stress, EVs are thought to have either direct or indirect effects differentially modulating reactive oxidative species (ROS) metabolism, depending on the physiological conditions (Bodega et al., 2019). In fact, epithelial EVs derived from acute coronary syndrome patients indirectly induced oxidative stress and premature endothelial senescence in the recipient cells (Abbas et al., 2017). Moreover, platelet-derived EVs from septic patients directly enhanced cellular stress and subsequent apoptosis in endothelial and vascular smooth muscle cells *via* NADPH oxidase (Janiszewski et al., 2004). In contrast, direct antioxidative potential has been reported for EVs loaded with various antioxidant enzymes, like superoxide dismutase 2, catalase or glutathione peroxidase, serving as ROS scavengers (Bodega et al., 2019). Indirect antioxidative effects were described in case of MSC-EVs, which alleviated harmful effects of acute kidney injury by enhancing activation of nuclear factor E2-related factor 2 (Nrf2), the key transcription factor of antioxidant responses (Zhang et al., 2016a).

After trauma, there is also a local and systemic complement activation as a major fluid phase innate immune system (Huber-Lang et al., 2015; Chakraborty et al., 2018). Due to the microenvironmental changes (e.g., ROS, DAMPs, proteases, and coagulation factors), the complement cascade is rapidly activated and generates anaphylatoxins, opsonins and membrane attack complexes (MAC) (Ricklin et al., 2010; Chakraborty et al., 2018). Concerning interactions of shedded vesicles with complement, there is increasing evidence that these circulating vehicles contain key complement factors, as well as complement regulators on their surface, thereby modulating the pro- and anti-inflammatory balance of the immune response and associated inflammation (Karasu et al., 2018). Furthermore, the EVs themselves can regulate complement activation, e.g., by harboring complement receptor 1 (CR1), CD55, and CD59 (Karasu et al., 2018). Overall, EVs may appear in a Janus-faced manner, on one hand protecting cells from MAC-driven cell lyses by shedding off the MAC from the cell surface, and on the other hand increasing cellular susceptibility by shedding off CD59 as major MAC inhibitor.

Taken together, EVs are involved in modulation of several trauma-related pathomechanisms, promoting both harm-reducing, but also detrimental effects; mainly depending on the donor cell and physiological conditions.

### EVs in Osteoarthritis (OA)

OA is a leading cause of disability and source of societal cost in older adults. It is a whole-joint disease in which all components of the joint are affected, involving structural alterations in the articular cartilage with additional abnormalities in subchondral bone, ligaments and synovium. During the development of OA pathology, the composition, functional properties, and structures of these tissues undergo marked alterations. Although pathological processes might selectively target a single joint tissue, ultimately all of the components are affected because of their intimate association (Loeser et al., 2012). With an aging and increasingly obese population in western countries, OA is becoming even more prevalent than in previous decades. OA accounted for 3.9% of years lived with disability worldwide in 2015, and by 2020 it is expected to be the fourth leading cause of years lived with disability globally (Hunter et al., 2014).

EVs may critically contribute to disease progression in OA. For example, it has been shown that EVs from IL-1ß-stimulated synoviocytes, mimicking the inflammatory joint environment in OA, displayed matrix remodeling activity as discussed before (see section Matrix Remodeling and Mineralization) and induced proteoglycan release from cartilage explants. Inflammatory cytokines, IL-6, MMP-3 and VEGF in EVs were only present at a low level and IL-1ß, TNF-α, MMP-9, and MMP-13 were not detectable in those EVs. In total, 50 miRNAs were differentially expressed in EVs from IL-1ß stimulated synoviocytes compared to non-stimulated cells (Kato et al., 2014)

As mentioned before EVs might also qualify as biomarkers. A recent microarray analysis of miRNAs in EVs extracted from synovial fluid showed that in samples from OA patients miRNAs are differentially regulated in a gender specific manner (Kolhe et al., 2017). The most differentially regulated GO biological processes by up-regulated miRNAs in males are cell-cell signaling, immune system process, response to stress, and a number of TLR signaling pathways, whereas in females cellular lipid metabolic processes, mitotic cell cycle and clathrin-sculpted monoamine transport vesicle membrane signaling is up-regulated. The most differentially regulated GO biological processes by down-regulated miRNAs in females are cell-cell signaling, immune system processes, innate immune response and number of TLR signaling pathways, whereas in males various metabolic processes (such as glycosaminoglycan, chondroitin sulfate, phospholipid, and keratan sulfate), ECM organization and cellular component assembly are reduced. These observations might explain a prevalence of OA in females. Based on the current knowledge of EVs from synovial fluid (SF) and serum of patients with OA, at least three possible diagnostic and prognostic EV biomarker types could be defined for joint diseases. (1) Immune-cell-derived inflammatory EVs in the circulation could be a sign of early-onset joint disease; (2) EVs in the SF of patients could provide information about inflammation type, disease state and progression; (3) EVs derived from synovial fibroblasts and chondrocytes, isolated from healthy, non-OA affected individuals, could provide information about predisposition for cartilage disorders (Malda et al., 2016).

## MSC as a Source of EVs—Influence of “MSC State” on EVs

In the past years, many therapeutic approaches for musculoskeletal disorders have been focused on MSC-based cell therapies due to their differentiation and immunomodulatory properties. MSC-EVs may account for a large part of these functions and are therefore under investigation as an alternative therapeutic approach to cell therapies. Application of EVs can circumvent some drawbacks of cell-based therapies, as discussed before (Toh et al., 2018). Successful clinical application of MSC-EVs has already been described for the treatment of graft-versus host disease (Kordelas et al., 2014).

### MSC Source and Culture Conditions

It is well-established that MSC populations from different tissue sources are not identical but exert tissue-specific characteristics and functions (Sacchetti et al., 2016). Next to differences between MSCs from different tissues and anatomical localizations, isolation procedures and *in vitro* culture itself may also change the phenotype of MSCs (Bara et al., 2014; Herrmann et al., 2016, 2019; Haddouti et al., 2020; Walter et al., 2020). Similarly, donor characteristics such as age and sex, as well as co-morbidities have been suggested to result in differences in MSC populations (Muschler et al., 2001; Dexheimer et al., 2011; Cassidy et al., 2020). Since it is generally assumed that the EV composition mirrors the characteristics of the originating cell population, aforementioned variabilities are likely to be transferred to the resulting EV preparation. Indeed, it was demonstrated that depending on the MSC source, the function and phenotype of MSC-EVs vary profoundly (Borger et al., 2017). Using RNA sequencing and comparative analysis, it has been shown that EVs derived from MSCs of human adipose tissue (AT) *vs*. bone marrow present a significantly different composition of their content. These reports suggest that intrinsic differences among MSC-EVs isolated from different tissue sources need to be considered as these differences may have a profound impact on EV-activity and therapeutic outcomes (Mianehsaz et al., 2019). These findings clearly indicate that MSC-EV-based therapies need to be adapted and optimized for the intended therapeutic application. Besides microenvironmental influences on the MSCs (see section Influence of Pathophysiologic Environments on MSC-EVs), further considerations are the choice of an allogenic or autologous setting as well as scalability of the production procedure when large EV quantities have to be produced, which has been recently discussed by Adlerz et al. (2020).

Moreover, several parameters of the EV preparation procedure have been reported to impact on EV yield and composition (Witwer et al., 2019), which is of particular importance for production of EVs for clinical application (see also section Considerations for Clinical-Grade MSC-EVs). It has been shown that cell seeding density may determine EV yield, with lower seeding densities favoring higher EV production rates. This study further suggested that shorter media collection periods might further enhance EV yields, while MSC passage did not account for significant differences (Patel et al., 2017). EV yields might be of critical importance, especially when it comes to therapeutic application. Thus, it should be noted that dynamic cultivation methods and/or bioreactor systems have been suggested to improve EV yields in different cell types (Yan et al., 2018).

Composition of cell culture media is a major determinant for the resulting EV preparation. In particular, fetal bovine serum (FBS), commonly used as nutrient source in MSC cultures contains high numbers of EVs itself, which unavoidably will contaminate the EV preparation. Human platelet lysate (hPL) is a well-established autologous alternative to FBS (Lang et al., 2018), yet it might lead to proteomic changes in MSCs which will most likely also be transmitted to EVs collected from these cells (Loibl et al., 2016; Lang et al., 2017). Nevertheless, hPL supplementation has been successfully used for EV production, even for clinical application (Kordelas et al., 2014). Various strategies have been established to avoid co-precipitation of EVs from culture supplements as discussed below (see section Isolation and Enrichment Protocols).

Thus, standardization of the isolation, characterization, and expansion of MSCs is of utmost importance in the production of EVs (Thery et al., 2018; Witwer et al., 2019).

### Influence of Pathophysiologic Environments on MSC-EVs

#### The Impact of Senescence and Age-Related Changes

Chronic inflammation in the elderly is described as a main contributor to reduced regenerative capacity of the skeleton. Inflamm-aging is induced by a pro-inflammatory cytokine milieu, which can result from oncogenesis or BMSC driven skewing of hematopoiesis toward the myeloid lineage, and these effects are to a certain extent mediated by MSC-EVs (Lee and Yu, 2020).

Recent studies in mice demonstrated a critical influence of inflamm-aging on skeletal stem cells and it can be assumed that similar effects may occur in human MSCs, and eventually also affect MSC-EVs. In this context, it was shown that skeletal stem/progenitor cells (SSPCs) from young mice (3 months) exposed to sera from middle-aged mice (13 months) developed a reversible age-associated cytokine profile, subsequently leading to a systemic pro-inflammatory environment. This study pointed out the possibility to restore SSPC potential by abrogating their senescent profile using rejuvenating anti-inflammatory treatments (Josephson et al., 2019). In addition, BMSC-EVs might have significant age-dependent differences in their immune activities. Namely, BMSC-EVs from the adult group contained the highest level of miR-335, associated with cell senescence and, while “younger” BMSC-EVs contained the highest level of miR-21, involved in negative regulation of macrophage-induced inflammation (Fafian-Labora et al., 2017). Although, “young” and “aged” human AT-MSC-EVs shared similar phenotype features, only “young” MSC-EVs showed protective roles in LPS-induced lung injury in a mouse model where these EVs induced M2 polarization in macrophages and reduced macrophage recruitment. This was associated with higher expression of miR-223-5p and lower levels of miR-127-3p and miR-125b-5p in “young” MSC-EVs (Huang et al., 2019). EVs isolated from early-passage human MSCs (about 15 population doublings, PD15) and late-passage MSCs (PD40) differently regulated immune cell activity. PD15 MSC-EVs were more effective than PD40 EVs in suppressing the secretion of Th1 and Th17 cytokines, as well as IFN-γ, in stimulated mouse splenocytes, while the effects of their EVs on the secretion of TNF-α and IL-6 in stimulated splenocytes were similar. Upon administration in LPS-challenged mice, only early-passage MSC-EVs showed immunosuppressive potential, where proteomic data revealed that early-passage MSC-EVs are enriched in TGF-βI, PTX3, EDIL, BGN, and LUM proteins (Kim et al., 2020).

Emerging evidence indicates EVs as important mediators of SASP effects, capable of transmitting paracrine senescence to nearby cells by regulating cell proliferation, and inflammation (Takasugi, 2018; Borghesan et al., 2020). It was shown that when “young” murine BMSCs endocytosed EVs derived from aged marrow, this led to reduction of osteogenic differentiation and proliferation in “young” MSCs, increasing their senescence (Davis et al., 2017). On the other side, purified induced pluripotent stem cell (iPSC)-EVs added to senescent MSCs showing high level of ROS production could reduce cellular ROS levels and alleviated aging phenotypes of senescent MSCs. This effect was mediated by delivery of intracellular antioxidant enzymes, PRDX1 and PRDX2 (Liu et al., 2019). Although certain potential of EVs from young individuals to attenuate senescence-related cell damage was observed, additional experiments are needed to completely reveal the rejuvenating potential of MSC-EVs (Melidoni, 2020).

Collectively, understanding of senescence program heterogeneity, together with permanent interaction with immunity, might improve current approaches for targeting senescent cells by senolytics, senostatics, or senogenics. Elucidation of EV contents and mechanisms of paracrine senescence would be of interest to set up optimal diagnostic and therapeutic strategies (Jeon et al., 2019; Kirschner et al., 2020). Particularly, improved characterization of aged cell-derived EVs would allow a better prediction of EV-derived effects achieved in healthy (incidental) and diseased (targeted) habitats. Also, conduction of *in vivo* and human cell-based studies in the future will reveal the translational relevance.

#### The Impact of a Diseased Environment

In addition to inflammation and age-related changes in MSC discussed before, it has also been suggested that their phenotype is affected by specific disease environments, such as osteoporosis, or OA (Egermann et al., 2010; Benisch et al., 2012; Camernik et al., 2020). Based on the evidence that the protein cargo of EVs reflects the pathophysiological status of the parental cells (Mianehsaz et al., 2019), it can be assumed that these traits are transferred to the derived EVs, and this has to be considered when choosing the MSC source for EV production.

Not much has been reported yet about the influence of a pathophysiological osteoporotic or OA microenvironment on the cargo of EVs. Nevertheless, analysis of cargos of EVs prepared from RA and OA patients clearly revealed differential expression of mRNAs, miRNAs, transcription factors, signaling molecules, and other proteins, according to disease entity. The cargo of EVs isolated from SF is different between OA patients and those without OA (Withrow et al., 2016). The content of the OA EVs revealed a several-fold increase in miR-200c, which is oxidative stress-sensitive and indirectly represses transcription factor ZEB1 that plays a role in maintaining articular cartilage homeostasis.

Collectively, these data demonstrate that not only the cellular origin of EVs needs to be considered, but also a potential pathophysiological *vs*. physiological state of the corresponding tissue.

### Alternative Cell Sources

#### iMP-EVs as an Alternative to MSC-EVs

The transcriptional and metabolic programs that define a “cell state” acquired during cell differentiation, tissue development, pathology, and age, are set by epigenetic modifications of the chromatin. Epigenetic reprogramming can revert these modifications along with the acquired “cell state” and rejuvenate cells to regain the cellular phenotype in the very early embryo (Takahashi and Yamanaka, 2006; Takahashi et al., 2007). Thus, the resulting reprogrammed iPSCs restore their unlimited proliferative potential and the capacity to differentiate into virtually any cell type (Tabar and Studer, 2014), including MSC-like mesenchymal progenitor cells (iMPs). Therefore, iPSCs-derived iMPs offer an opportunity to obtain large amounts of highly regenerative patient-specific EV-producing MSCs, independently of donor age, tissue source, or disease.

Different approaches for differentiation of iPSCs into iMPs have been reported frequently, including their subsequent downstream specification into mesoderm-derived lineages, such as chondrocytes, osteoblasts, and myocytes (Li et al., 2010; Guzzo et al., 2013; Koyama et al., 2013; Diederichs and Tuan, 2014; Frobel et al., 2014; Hynes et al., 2014; Kang et al., 2015). IMPs comply with the defining characteristics of *in vitro*-cultured MSCs (Dominici et al., 2006), as they express similar cell-surface markers: CD90, CD73, CD105, and CD44, but not CD45 and HLA-DR. Similarly to MSCs, they are capable of *in vitro* differentiation into osteogenic, chondrogenic, and adipogenic lineages (Lian et al., 2010; Hynes et al., 2014). One very interesting distinction from adult MSCs is that iMPs appear to maintain an increased juvenility, as they resemble rather fetal MSC than adult MSC characteristics according to RNA sequencing (Chan et al., 2018), gene expression (Buchert et al., 2019; Spitzhorn et al., 2019) and epigenetic data (Frobel et al., 2014). Whether this juvenility is also transmitted by the produced EVs and whether iMP-EVs can thus rejuvenate target cells and/or alleviate senescence would be interesting to investigate.

Surprisingly, iMP-derived EVs have not been investigated in full so far, with fewer than 15 studies reported to date (Hu et al., 2015a; Zhang et al., 2015b, 2016d; Nong et al., 2016; Qi et al., 2016; Du et al., 2017b; Liu et al., 2017a; Zhu et al., 2017; Kim et al., 2018; Fang et al., 2020). Therein, iMP-EVs attenuated limb ischemia in a mouse model, where treatment with iMP-EVs strongly reduced the incidence of limb loss and increased microvessel density (Hu et al., 2015a). Supportive mechanistic *in vitro* studies indicated that iMP-EVs could stimulate migration, proliferation, and angiogenesis-related gene expression of endothelial cells. Two subsequent studies from the same group have reported that bone repair could also be stimulated by iMP-EVs in a rat cranial head model, as well as iMP-EVs could enhance mineralization and ALP activity of MSCs cultured in osteogenic medium (Qi et al., 2016; Zhang et al., 2016d). Moreover, steroid-induced osteonecrosis in femoral heads of rats, as well as collagenase-induced osteoarthritis in mice, were reduced by iMP-EVs (Liu et al., 2017a; Zhu et al., 2017). While collectively these reports appear to be promising concerning the therapeutic effectivity of iMP-EVs for treatment of musculoskeletal disorders, an independent reproduction of these studies, as well as detailed elucidation of the mechanisms regulated by iMP-EVs, are urgently needed as the next step. Fundamental mechanistic and functional studies are required to identify the cargo of iMP-EVs and the key molecules responsible for their mode of action in specific *in vivo* settings. This will be essential for answering the questions whether iMP-EVs can elicit similar regenerative effects as transplanted MSCs, or can outperform MSC-EVs and therefore offer iMPs as an attractive alternative cell source of EV production for cell-free therapy. Identifying the therapeutically active EV components will also facilitate future experiments to modulate culture conditions and tailor iMPs to produce EVs with a specific cargo composition. For example, molecules supporting cartilage matrix production and suppressing matrix-degrading enzymes, as well as pro-inflammatory cytokines would be essential for cartilage regeneration, while stimulation of angiogenic, immunomodulatory, and osteogenic activities would be important for bone regeneration.

One hurdle yet to overcome is that iMPs are currently strongly heterogenic and variable, which severely limits the reproducibility of iMP studies (Diederichs and Tuan, 2014). More sophisticated differentiation strategies that use temporally varying cytokine cocktails to more stringently guide iPSC differentiation into the mesodermal and then the mesenchymal lineage have already brought first successes (Kreuser et al., 2020) and will in future help overcome this limitation.

**Figure 2:**
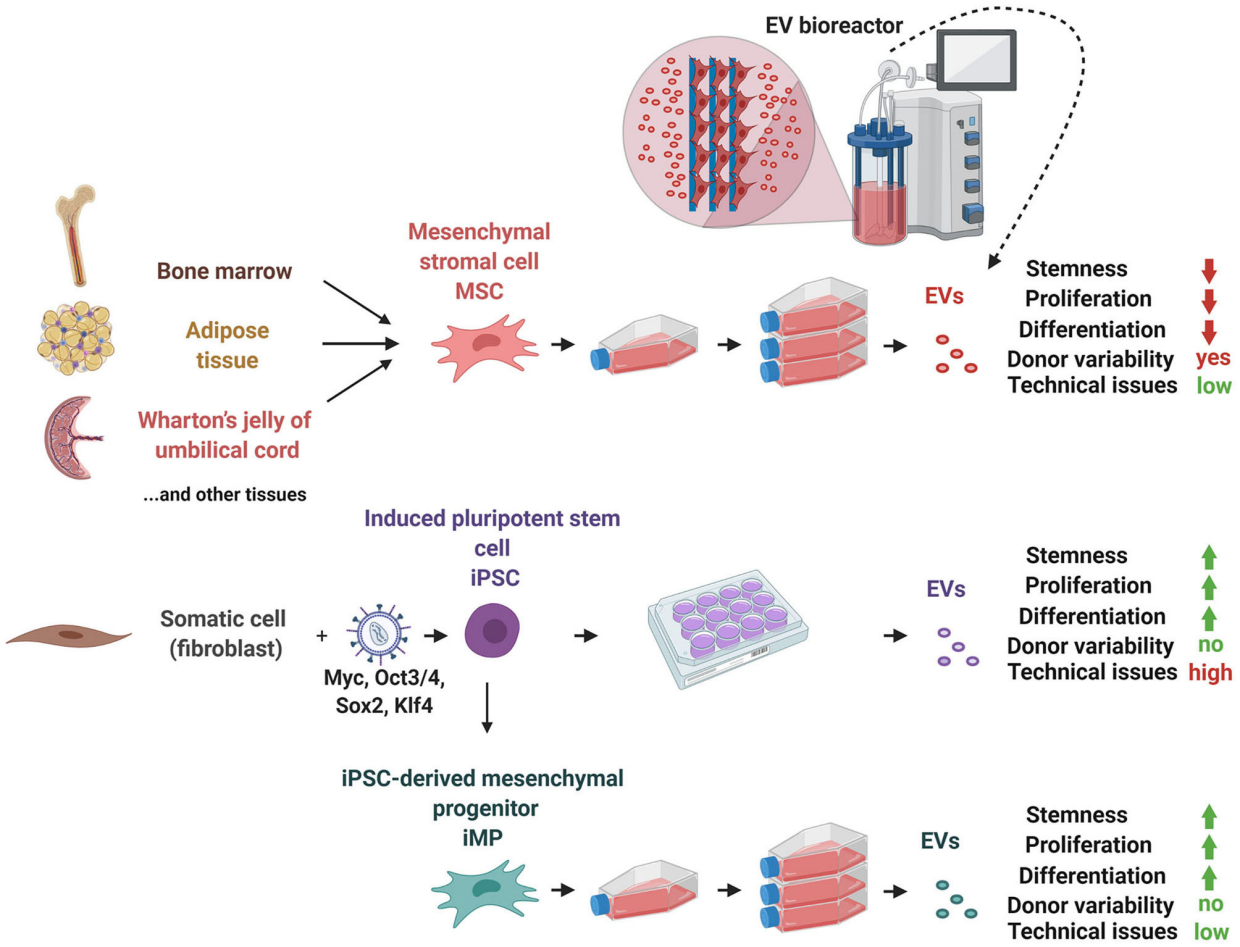
iMP as an alternative cell source of EVs. Although MSCs can easily be maintained in cell culture enabling the production of large volumes of EV-conditioned medium, their proliferation and tri-lineage differentiation capacity declines during prolonged expansion and they are affected by donor variability. Induced pluripotent stem cells (iPSC) that derive from a somatic cell that underwent reprogramming with Myc-Oct3/4-Sox2-Klf4-expressing lentivirus represent an alternative cell source, with unlimited proliferation and differentiation potential that can overcome the donor variability issue. However, these benefits are surpassed by the technical issues related to iPSC maintenance and expansion in culture, as they require expensive media supplements, meticulous day-to-day inspections and handling, which make iPSC expansion in order to increase iPSC-EVs yield quite a challenging and expensive task. Alternatively, iPSC could be subjected to transdifferentiation into mesenchymal progenitor cells (iMP). These cells possess combined characteristics of iPSCs and MSCs: on one hand, they could be easily expanded and maintained in cell culture, like MSCs, and on the other hand, they inherit and retain the high proliferation and differentiation capacities of iPSCs.

Taken together, iMPs are an inexhaustible and juvenile alternative cell source for EV production that holds a promise to overcome limited availability and activity of adult MSCs that are compromised due to age and underlying pathologies of human donors (Figure 2). While initial preclinical studies suggested potent and broad therapeutic effects comprised by iMP-EVs, follow-up detailed studies that would characterize high-quality iMP populations and their EVs are required to establish whether iMP-EVs could indeed fulfill these tremendous expectations.

#### Chondrogenic Stem/Progenitor Cells (CSPCs)

Some years ago, a small population of MSC-like progenitor cells have been described within the cartilage—the chondrogenic stem/progenitor cells (CSPCs) (Alsalameh et al., 2004; Fickert et al., 2004; Koelling et al., 2009). Due to rather strong similarities to BMSCs, it might be possible that CSPC-derived EVs represent another interesting player or therapeutic tool in the pathogenic scenario of OA. In fact, CSPCs are thought to be involved in cartilage regeneration as implied by enhanced migratory activity, proliferation and immunomodulatory features after cartilage trauma (Seol et al., 2012; Riegger et al., 2018). Moreover, it was recently shown that intraarticular application of EVs from murine chondrogenic progenitor cells (CPC) attenuated OA development in a murine destabilization of the medial meniscus (DMM) OA model (Wang et al., 2020). Interestingly, CPC-EVs from MRL/MpJ superhealer mice exhibited stronger therapeutic effects as compared to CPC-EVs from non-superhealer mice, which was ascribed to high levels of miRNA 221-3p (Wang et al., 2020).

## Practical Considerations For Ev-Based Therapies

### Isolation and Enrichment Protocols

**Figure 3:**
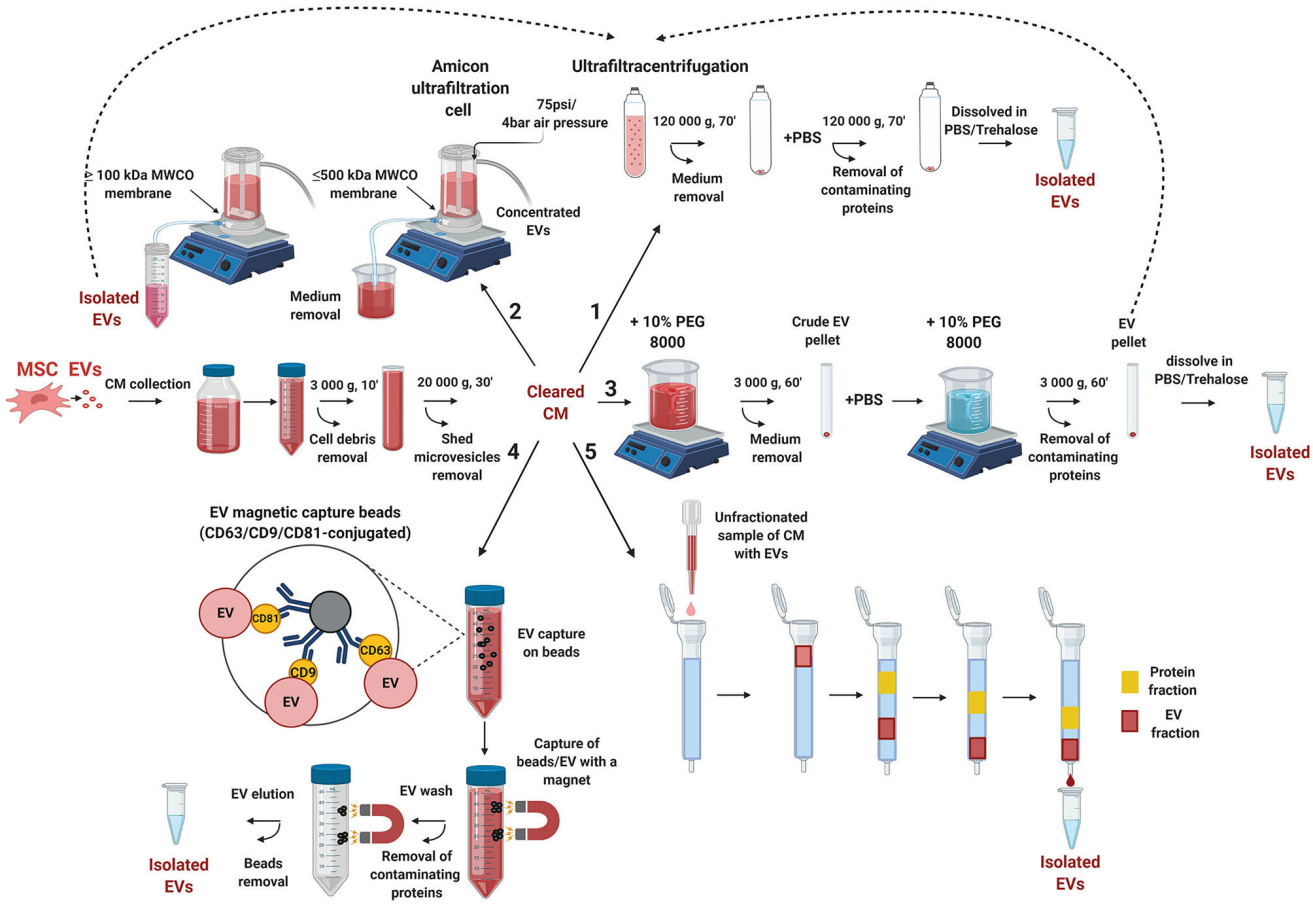
Overview of different methodological approaches for isolation and purification of EVs. MSC-EVs secreted into a cell culture supernatant are collected as EV-conditioned medium (CM). All EV isolation protocols start with sequential low-speed centrifugation steps, to remove cell debris and large shed microvesicles. Next, the cleared CM can undergo either ultracentrifugation (**1**), ultrafiltration (**2**), precipitation with PEG (**3**), immune-immobilization (**4**), or size-exclusion chromatography (**5)** protocols resulting in isolated EV fractions. (**1**) CM is subjected to ultracentrifugation at 120 000 × g. For removing contaminating proteins, the EV pellet is additionally dissolved in PBS, and the ultracentrifugation step is repeated. (**2**) In case of ultrafiltration, the CM is passed through a membrane with pore sizes over 500 kDa MWCO by air pressure application. This results in concentrated CM with reduced serum protein and lipoprotein content. Next, the concentrated CM can either be subjected to a next round of ultrafiltration, using a membrane with pores ≤ 100 kDa MWCO, to retain and capture EVs, or, alternatively, it can undergo the ultracentrifugation procedure (**1**). For the PEG protocol (**3**), EVs are precipitated from the CM by 10% PEG in two rounds. The final EV pellet can be additionally purified with subsequent ultracentrifugation steps. For the immunocapture protocol (**4**), CD9-, CD63-, or CD81-conjugated magnetic beads are incubated with CM, to capture EVs, which are then immobilized by a magnet. After serial washing steps, the captured EVs are eluted from the beads. In case of size-exclusion chromatography (**5**), CM, or concentrated CM, is loaded on a resin-filled column, and the CM content (EVs and medium proteins) is fractionated according to their size. This approach allows isolation of uniformly-sized EV particles. All methods can be used as separate protocols or in various combinations, to fulfill different requirements for downstream analyses and applications utilizing EVs.

Many different EV isolation techniques have been developed to date (Greening et al., 2015; Li et al., 2017; Monguio-Tortajada et al., 2019). They are based on ultracentrifugation (Momen-Heravi, 2017), ultrafiltration (Gupta and Marcela Rodriguez, 2019), size-exclusion chromatography (Lobb and Möller, 2017), immunoaffinity capture (Oksvold et al., 2015), polymeric precipitation (Weng et al., 2016; Ludwig et al., 2018), or microfluidic separation (Contreras-Naranjo et al., 2017) (Figure 3). All these different approaches have been developed to facilitate separation, purification, and concentration of EVs from contaminating cell debris and interfering components of cell culture media; as well as to enable purification of EVs from different clinical sample matrices: primary blood (Wu et al., 2017), urine (Street et al., 2017), amniotic fluid (Ebert and Rai, 2019), saliva (Lässer et al., 2012), etc. To facilitate isolation of specific EVs, each protocol exploits a particular property of EVs, such as their density, size, shape, or a specific set of protein markers.

#### Methodological Approaches to Reduce Contamination With Serum-Derived EVs and Serum Albumin From EV-Conditioned Medium

Regardless of a protocol selected for EV preparation, measures have to be taken to deplete the EV conditioned medium of serum albumin that otherwise hinder EV preparation, purification, and detection (Abramowicz et al., 2018; Lehrich et al., 2018). Additionally, FBS supplementation of cell cultures can lead to contamination of the EV preparation by serum-derived EVs, which may also affect *in vitro* cell behavior, as it has been shown for migration and proliferation (Shelke et al., 2014). There are commercially available EV-depleted serum sources (Kornilov et al., 2018), however, these are quite expensive. Researchers often try to overcome this by preparing their own EV-depleted serum. EV elimination protocols usually involve prolonged ultracentrifugation (up to 18 h at 100 000 × g) of diluted serum stocks (Shelke et al., 2014), or a preliminary ultrafiltration step of serum-containing media before it is usable for cell culture (Kornilov et al., 2018). However, extended ultracentrifugation can also lead to co-precipitation of serum albumin and lipoproteins reducing the capacity of the resulting media to support cell growth (Eitan et al., 2015). An alternative to this would be an ultrafiltration approach that utilizes commercially available filtration membranes with large pore sizes of 500 or 1,000 kDa MWCO (molecular weight cut-off) (Gupta and Marcela Rodriguez, 2019; He et al., 2019). These membranes allow all serum proteins to pass freely through but capture contaminating serum EVs. Such small volume size ultrafiltration devices (up to 14 ml load per device) are subjected to low-speed centrifugation, thus all the nutrients and serum proteins remain intact in the media, and cell growth is not affected detrimentally. An even better solution could be the application of the ultrafiltration approach without any centrifugation procedure using stirred cell units that rely on air compression to pass media through ultrafiltration membranes (Gupta and Marcela Rodriguez, 2019). These are available in large volume sizes, up to 400 ml, thus allowing large volumes of EV-free media preparations. Moreover, this approach could also be applied for removing serum proteins that interfere with EV purification and detection from EV-conditioned media before subjecting it to any of the EV isolation protocols. Since serum protein sizes range from ~ 67 (albumin, that comprises almost 50% of serum protein content) to ~ 900 kDa (IgM) (Issaq et al., 2007), ultrafiltration of serum-containing medium through 500–1,000 kDa MWCO membranes removes the interfering serum proteins from the conditioned medium, thus improves purity and detection of EVs (Benedikter et al., 2017).

#### Methodological Approaches for Isolation of EVs: Pros and Cons

For EV production, cells normally are grown in T flasks that enable collection of culture media conditioned with secreted EVs. However, this process has some limitations when a large input volume, high yield and concentration are required for EV mass-production. Additionally, the need to use FBS when cells are cultured in a conventional system, brings more challenges when EV manufacturing for clinical use requires homogeneous and efficient EV preparations. These obstacles could be overcome with application of bioreactors (Yan et al., 2018). Once the EV-conditioned media has been produced and collected, it undergoes different concentration, isolation and purification protocols. All the protocols for EV isolation usually start with several initial differential centrifugation steps, which are necessary to remove dead cells, cell debris, and contaminating apoptotic bodies. After this, the EV-conditioned media can undergo isolation protocols utilizing an ultracentrifugation approach, or are subjected to further concentration and serum albumin depletion steps, before EV purification (Figure 3).

##### Ultracentrifugation-Based Protocols (1)

Ultracentrifugation is the classical approach for the isolation of EVs (Momen-Heravi, 2017), in which the centrifugal force used typically ranges from ~100 000 to 120 000 × g. The formed pellet contains a crude EV preparation, and, for further purification, the pellet is dissolved in PBS (phosphate-buffered saline), and the ultracentrifugation step is repeated. There are variations of the classical ultracentrifugation protocol. To achieve a pure EV preparation, with particles of uniform size, a sample can be layered onto the top of density gradient medium (commonly, sucrose is used for the formation of step gradients, and Percoll is applied to form continuous gradients) and subjected to an extended round of ultracentrifugation (Tauro et al., 2012; Momen-Heravi, 2017; Pérez-González et al., 2017). The separated EVs can then be recovered by simple fraction collection. A downside of a density gradient based ultracentrifugation protocol is that its capacity is limited by the narrow load zone. Additionally, fraction collection and recovery might result in a substantial reduction of the EV yield. Although the ultracentrifugation method is overall robust and has low running costs in terms of necessary consumables and reagents, it requires substantial investments in the initial equipment. Other limitations of the protocol are a long running time, especially when large volumes of conditioned media need to be processed, and it is labor intensive. Moreover, high-speed centrifugation may result in EV damage, and, if the density gradient step is omitted, the EV preparation is often of low purity impeding downstream analyses (Lamparski et al., 2002; Taylor and Shah, 2015).

##### Ultrafiltration-Based Protocols (2)

To reduce the time for processing large starting volumes of EV-conditioned media, and to remove contaminating serum proteins, and lipoproteins that might co-precipitate with the EVs, additional measures could be taken. As mentioned above, large volumes of starting media could be reduced using the ultrafiltration devices that utilize membranes of pore sizes of 500 or 1,000 kDa MWCO. EVs' size distribution between 50 and 150 nm would prevent them from passing through these membranes, and excess of medium with all the contaminating serum proteins is thus successfully removed (Gupta and Marcela Rodriguez, 2019).

##### Polymer Precipitation-Based Protocols (3)

Another approach to isolate EVs is based on their precipitation together with polymers, such as polyethylene glycol (PEG) (Weng et al., 2016; Ludwig et al., 2018). PEG alters the solubility of EVs as it extrudes water from the solution. The aqueous PEG wraps EVs together, thereby forming EV aggregates which can be then precipitated by a low-speed centrifugation step. This method is very cost-effective, as it does not require expensive equipment or reagents. It also can be applied for the isolation of EVs from biological fluids, such as whole blood, plasma, urine, etc. (Rider et al., 2016; Lv et al., 2018). The downside of this protocol is that other non-EVs contaminants, such as proteins and polymeric materials, could co-precipitate, thus pre-and post-clean-ups are usually needed, including ultracentrifugation and ultrafiltration steps that were mentioned before.

##### Size Exclusion Chromatography-Based Protocols (4)

A popular size-based separation technique for EV isolation and purification is size exclusion chromatography (SEC) (Lobb and Möller, 2017). For this, a porous material to sort particles of a defined size is used as an immobilized phase in a column, and EV-containing medium is passed through the column, to separate all the macromolecules and particles according to their size. This method results in structurally intact and size-unified EVs with high purity and integrity, thus SEC is especially in demand for EV applications for immunosuppressive effects (Monguio-Tortajada et al., 2017, 2019). However, the downside of this approach is a low sample capacity, high running costs, and it is time-consuming. Additionally, this method is not suitable when the preparation yield needs to be scaled up, and it requires dedicated expensive equipment.

##### Magnetic Beads Immune-Immobilization-Based Protocols (5)

This method used for EV isolation involves microbeads coated with antibodies to certain protein markers present on the EV surface (Oksvold et al., 2015; Poellmann et al., 2020). The advantage of this technology is that it can isolate EVs from any type of fluids. In this method, commercial magnetic beads are mixed with the EV-containing sample, and, after short incubation, the immobilized EVs are separated from the rest of the medium by a magnet. After washing, EVs can be recovered from the beads. For this, solutions containing SDS (sodium dodecyl sulfate) or high salt concentrations, or glycine-based low pH buffers can be used. However, depending on downstream applications, neither of these elution buffers might be suitable, as it can result in capture antibodies leaching, or EV loss due to their incomplete recovery from the beads. Nevertheless, the advantage of this isolation method is its ability to select a specific EV population based on marker expression regardless of EV size. But with this comes the main limitation, as there is no universal EV marker defined to date that is uniformly present in all EVs. It also requires expensive reagents, as well as it has a low capacity and yields that would be further decreased in a case when an antigenic epitope is blocked or masked. Side-by-side comparison of several commercially available kits for EV immunocapture demonstrated that to achieve sufficient purity of the EV preparation, additional washing and ultracentrifugation steps would be still required that might also compromise its time and yield efficiency (Patel et al., 2019).

In conclusion, despite of recent progress and advances in the methodological approaches addressing EV isolation and purification, no ideal universal method suitable for all applications and tissue and cell types has been developed yet. However, with many to date available approaches, there is a strong fundament for researchers to adapt EV preparation procedures to certain experimental needs.

### Quality Control

EV preparations are subject of great heterogeneity due to variability of the originating cells (see section MSC as a Source of EVs—Influence of “MSC State” on EVs) and alterations arising from different isolation strategies. However, for deciphering the functional relevance of EVs as well as the development of efficient therapies with translational success, standardization is required. Minimal criteria for definition and classification of EVs along with recommendations for appropriate analyses have therefore been published by the International Society for Extracellular Vesicles (ISEV) in 2014 (Lotvall et al., 2014) and updated in 2018 (Thery et al., 2018). Most commonly applied measurements are briefly summarized below.

#### EV Visualization

SEM (scanning electron microscope) and TEM (transmission electron microscope) are recommended to characterize the morphology and distribution of EVs (Wu et al., 2015). Using SEM, EVs isolated from MSCs exhibit a round morphology and uniform distribution in size. On the other hand, TEM is considered as a standard tool for characterizing the morphology of EVs. Observed by TEM, MSC-EVs show a typical “cup-shape” morphology.

#### EV Size Distribution and Concentration

Light scattering technologies, for example, NTA (nanoparticle tracking analysis) are frequently applied to determine the size distribution and concentration of EVs, alternatively tunable resistive pulse sensing (TRPS) can be used. Additionally, standard flow cytometry and high resolution flow cytometry are used for larger or smaller EVs, respectively (Atkin-Smith et al., 2015; Maas et al., 2015).

#### EV Protein Marker

Flow cytometry and Western blot analyses are commonly used to identify membrane protein markers of EVs. For example, the transmembrane proteins (CD9, CD81, and CD63) and accessory proteins (Alix, TSG101, HSP70, and HSP 90) are valuable as biomarkers of EVs (Doyle and Wang, 2019). However, some proteins, which are not constituents of EV structures, are usually co-isolated with EVs in the procedure of ultracentrifugation. Therefore, GM130, calnexin, apolipoproteins A1/2, and B (APOA1/2, APOB), and albumin were reported as negative protein markers for EV preparations to assess the purity of EVs (Sódar et al., 2016; Karimi et al., 2018).

### Considerations for Clinical-Grade MSC-EVs

There is great interest and research effort in translating MSC-EV-based therapies into clinical application. Production of clinical grade EVs however requires highest standards of standardization and reproducibility, where variabilities on the level of parental cells as wells as culture conditions have a critical impact as also discussed above (see section MSC as a Source of EVs—Influence of “MSC State” on EVs). These hurdles have been recently recognized and addressed in a joint position paper by members of the Society for Clinical Research and Translation of Extracellular Vesicles Singapore (SOCRATES), the International Society for Extracellular Vesicles (ISEV), the International Society for Cell and Gene Therapy (ISCT) and the International Society of Blood Transfusion (ISBT) (Witwer et al., 2019). The paper suggests to build guidelines for MSC-EVs on both, the MISEV criteria for EVs (see sections Quality Control, EV Visualization, EV Size Distribution and Concentration, and EV Protein Marker) and the definition of human MSCs established by the Mesenchymal and Tissue Stem Cell Committee of the ISCT (Dominici et al., 2006). In addition, it has been recommended to specify the source of MSCs, the age and passage of MSCs and to conduct functional testing of MSC-EVs to verify the capability of MSCs in producing functional EVs. Because different protocols for purification of EVs may lead to different results, including the purity and yield, it is critical to define the standard isolation of EVs as described above and it needs to be recognized that it is likely that each MSC-EV preparation leads to a different product. The challenges related to the clinical translation of MSC-EVs have also extensively been discussed in a recent article (Maumus et al., 2020).

## Therapeutic Application OF MSC-EVs In Musculoskeletal Disorders—Where are We?

### Immunomodulation

Almost all musculoskeletal diseases have an immunogenic component that could benefit from an immune modulatory therapy, leading to a great interest of MSCs in musculoskeletal research in the last couple of years. Therapies using MSC-EVs could show promising results comparable to current treatment regimens with MSCs but without the downsides that accompany the usage of MSCs. Therefore, MSC-EVs could be a promising single or adjuvant therapy for musculoskeletal diseases.

RA has been a particular focus in the context of EV research. Current research suggests that EVs could be a novel avenue for the immune therapeutic treatment of RA, an overview on *in vivo* studies testing EV-based therapies for RA is presented in Table 1. Casado et al. (2017) showed that EVs from BMSCs may reduce antigen-specific T cell responses in an antigen-induced synovitis RA model. In detail, associated leukocyte infiltration and TNFα production were significantly reduced by injecting MSC-EVs directly into the affected joint (Casado et al., 2017). In line with these results, another *in vivo* study using murine models of delayed-type hypersensitivity and collagen-induced arthritis showed that EVs protected joint integrity by mediating anti-inflammatory effects (Cosenza et al., 2018). Interestingly, Skriner et al. (2006) showed that EVs from RA patients contained citrullinated proteins. These proteins are known autoantigens and biomarkers for RA and the fact that they can be found in EVs underlines a potential role of EVs in the immune-relevant distribution of citrullinated proteins. In line with this, another study found that the amount of platelet-derived EVs might be associated with RA activity (Knijff-Dutmer et al., 2002).

**Table 1:** Summary of preclinical studies testing the therapeutic application of MSC-EVs in musculoskeletal disorders.

**References**	**Animal model**	**Cell source, EV isolation**	**Administration, dosage**	**Follow-up**	**Effects, underlying mechanisms**
**EV therapy in rheumatoid arthritis (RA)**					
Casado et al. (2017)	Antigen (BSA)-induced synovitis RA model (pig)	Porcine iliac crest BMSCs; Ultrafiltration (UF)	Intra-articular (i.a.) injections of MSC-EVs; 500 μg protein per injection in 500 μL	7 days after i.a. injections	Leukocyte infiltration and TNFα production were significantly reduced
Cosenza et al. (2018)	Murine models of delayed-type hypersensitivity (DTH) and collagen-induced arthritis (CIA)	Murine BMSCs, C57BL/6; Ultrazentrifugation (UZ)	Fat pad injection for DTH, intra venous (i.v.) for CIA; 250 ng	DTH: day 6; CIA: until day 37	DTH: dose-dependent anti-inflammatory effect; CIA: decreased clinical signs of inflammation, fewer plasma blasts, and more Breg-like cells
Headland et al. (2015)	K/BxN model of arthritis and glucose-6-phosphate isomerase (GPI)-induced arthritis model (both mouse)	Human RA-derived synovial fluid; human RA neutrophils and macrophages; mouse bone marrow; UZ	i.a. injection of MV (3 ×10^4^ particles in 5 μL PBS) at day 3 after arthritis induction (K/BxN model); i.a. injection of MV (3 ×10^4^ particles in 5 μL PBS) at day 21 after GPI arthritis induction	• K/BxN model: after 5 days • GPI-model: after 25 days	MV treatment prevented GAG loss in knees in both exp. arthritis groups.
**EV therapy in traumatic joint injuries and osteoarthritis (OA)**					
Cosenza et al. (2017)	Collagenase-induced knee OA (mouse)	Murine BMSCs; UZ	i.a. injections at day 7 after OA-induction. Injection of MVs (500 ng/5 μL) or Exos (250 ng/5 μL)	42 days after OA-induction	• In MV/Exo groups: Protection of cartilage (less degradation) and reduction in osteophyte formation. Epiphyseal bone: higher BV/TV and less degradation in MV/Exo groups. • SB: as above and additional less calcification of ligaments and menisci
Mao et al. (2018)	Collagenase-induced knee OA model (mouse)	Human miR-92a-3p-overexpressing BMSCs; UZ	i.a. injections at day 7, 14, and 21 after OA-induction; 15 μL Exos (500 μg/mL)	28 days after OA-induction	WNT5A inhibition via miR-92a-3p; attenuated progression of early OA/reduced cartilage damage
Zhu et al. (2017)	Collagenase-induced knee OA model (mouse)	Human iPSC-MSCs = iMSCs (cell line CIP33), synovial membrane-derived MSCs (SMMSC); UF	i.a. injections at days 7, 14, and 21 post collagenase injection. 8 μL Exos (conc. 1.0 ×10^10^/mL in PBS) from both cell types	28 days after OA-induction	The OARSI scores in the iMSC-Exos, and SMMSC-Exos groups were significantly lower than in the OA group. ICRS scores revealed no significant differences among the normal, iMSC-Exos, and SMMSC-Exos groups but higher than the OA group. Best results in the iMSC-Exos group.
Ni et al. (2019)	Surgical destabilization of the medial meniscus (DMM) (mouse)	Human OA articular chondrocytes; UZ and UF	i.a. injection of Exo-like vesicles pre-treated with IL1ß (10^9^ particles in 5 μL = ChC^ILpre^) combined with antagomiR (5 nM in 5 μL) biweekly for 4 weeks, starting 4 weeks post-surgery	8 days post-surgery	i.a. injection of ChC^ILpre^ Exos aggravated cartilage erosion and synovitis in DMM induced OA mice, while antagomiR-449a-5p could partially reverse Ch^CILpre^-Exo-mediated cartilage damage
Tao et al. (2017)	DMM + anterior cruciate ligament transection (ACLT) (rat)	Human miR-140-5p-overexpressing synovial MSCs	i.a. injections, from the 5th to the 8th week post-surgery (weekly); 100 μL (10^11^ Exos/mL)	12 weeks post-surgery	Deceleration of OA progression (reduced joint wear and ECM loss/no type I collagen expression); miR-140-5p-mediated maintenance of SOX9 via inhibition of the Ras-like GTPase RalA and subsequent recovery of ECM secretion.
Wang et al. (2017)	DMM (mouse)	Human embryonic stem cell-induced mesenchymal stem cells (ESC-MSCs); UZ	i.a. injections of 5 μL (1 ×10^6^) EVs every 3 days, starting 4 weeks post-surgery	8 weeks post-surgery	Lower OARSI score; increased expression of type II collagen and decreased expression of ADAMTS5
Woo et al. (2020)	DMM (mouse)	Human adipose-derived stem cells (hASCs); size-fractionated/tangential flow filtration (TFF)	i.a. injections of 6 μL (1 ×10^8^) hASC-EVs every week; starting 5 weeks post-surgery	11 weeks post-surgery	Improved histological properties and lower OARSI score; reduced protease activity and percentage of MMP-13-positive chondrocytes; possibly mediated through miR-199a, miR125b, miR-221, and miR-92a
Chen et al. (2019)	Osteochondral defect; 4 mm diameter, 4 mm depth (rabbit)	Human BMSCs; UZ	3D printed ECM/GelMA/Exo scaffold; 200 μg/mL	6 and 12 weeks post-surgery	Enhanced M2 polarization of synovial macrophages, reduced fibrocartilage formation, restored chondrocyte mitochondrial function (mitoprotection)
Liu et al. (2017b)	Osteochondral defect; 4 mm diameter, 3 mm depth (rabbit)	Human iPSC-MSCs (iPS-S-01); UZ	acellular tissue patch (o-nitrobenzyl alcohol moieties modified hyaluronic acids/gelatin); 1 ×10^11^ mL^−1^	12 weeks post-surgery	Seamless integration with native cartilage matrix; hyaline cartilage formation
Sabry et al. (2018)	Osteochondral defect (3 mm diameter and 1 mm depth)/lateral para-patellar sti?e (mongrel dogs)	Canine BMSCs; UZ	i.a. injection of 100 μg MVs in 100 μL PBS	6 weeks, 3 and 6 months post-surgery	Increased healing of chondral defect over time, more chondrocytes, more collagen formation, increased defect filling
Wong et al. (2020)	Osteochondral defect; 4.5-mm diameter, 1.5-mm depth (rabbit)	Immortalized human ESC-derived MSCs (E1-MYC 16.3); TFF	i.a. injections at day 0, 7, and 14 post-surgery; 200 μg Exos suspended in 1 mL 3% (w/v) HA	6 and 12 weeks post-surgery	Improved biomechanical and structural properties compared to HA-treated defects; hyaline cartilage formation
Zhang et al. (2016d)	Osteochondral defect; 1.5-mm diameter, 1-mm depth (rat)	Human embryonic stem cell line (hESCs; HuES9); TFF	i.a. injections of 100 μL (100 μg Exos); directly post-surgery, then weekly	6 and 12 weeks post-surgery	Improved histological scores and complete neotissue filling of defect (5 out of 6: hyaline cartilage); reduced fibrous tissue formation
Zhang et al. (2018b)	Osteochondral defect; 1.5 mm diameter and 1 mm depth (rat)	Immortalized human ESC-derived MSCs (E1-MYC 16.3); TFF	i.a. injections of 100 μL (100 μg Exos); directly post-surgery, then weekly	2, 6, and 12 weeks post-surgery	Increased cellular proliferation/reduced apoptosis; enhanced matrix synthesis (type II collagen); regenerative immune phenotype (enhanced M2/decreased M1 macrophages); lower IL-1ß and TNF-α levels; overall improved repair of osteochondral defects
**EV therapy in fracture healing**					
Furuta et al. (2016)	Femur fracture model of male CD9-deficient mice	Human BMSCs and human osteosarcoma cell line (HOS); UZ	Local injection into the fracture part at days 1 and 8 post fracturing; 100 μL of concentrated Exos isolated from 2 mL BMSC culture medium	0, 1, 2, 4, and 6 weeks post fracture setting	BMSC Exos rescued delayed fracture healing in CD9-deficient mice; Exos promoted fracture healing (earlier bone union) in WT mice; HOS Exos had no positive effects on fracture healing
Li et al. (2018)	Skull defect model (4 mm critical sized hole) in male BALB/C mice	Human adipose derived stem cells (ASCs); UZ	Cylindrical PLGA scaffolds immersed in 1 μg/μL Exos solution (250 μL/scaffold) implanted into the skull defect	6 weeks after implantation	Increased new bone formation along the border and in the center of the defects; increased collagen formation; increased engraftment of host MSCs
Qi et al. (2016)	5 mm critical sized calvarial bone defects in ovariectomized rats (osteoporosis model)	Human iPSC-MSCs; UZ	ß-TCP scaffolds loaded with 100 μg/mL or 200 μg/mL Exos	2, 4, 6, 8 weeks after implantation	Increased BMD and BV/TV; increased neovascularization (increased vessel area and number); increased percentage of new bone formation area
Xie et al. (2017)	Male nude mice, subcutaneous bone formation model	Rat BMSCs; UZ	EV-modified decalcified bone matrix (DBM)-scaffolds (coated with 20 μg EVs plus MSCs alginate solution) were implanted subcutaneously	1 and 2 months after implantation	EV-modified DBM scaffolds enhanced bone regeneration in the central region of the grafts. A higher average numbers of CD31-positive vessels indicate promotion of vascularization

*BMSCs, bone marrow-derived mesenchymal stem cells; WT, wild type; iPSC, induced pluripotent stem cells; iMSCs, induced Mesenchymal Stem Cells; ESCs, Embryonic Stem Cells; ECM, extracellular matrix; PLGA, poly(lactic-co-glycolic acid); HA, hyaluronic acid; GAG, glycosaminoglycans; Exo(s), exosomes; EV, extracellular vesicles; MV, matrix vesicles; BMD, Bone Mineral Density; BV/TV, Bone Volume/Trabecular Volume; SB, Subchondral Bone*.

In the context of OA, the beneficial immunomodulatory properties of MSC-EVs have been confirmed as well. In the study of Tofino-Vian et al., EVs isolated from human AT-MSCs exerted a protective effect on chondrocytes from OA patients through multiple mechanisms, such as reducing the production of inflammatory mediators (e.g., TNF-α, IL-6, PGE2, and NO), decreasing total MMP activity, and enhancing the production of the anti-inflammatory cytokine IL-10 (Tofino-Vian et al., 2018).

In summary, EVs have a profound effect on a variety of immune processes and thereby contribute to health and disease in the musculoskeletal apparatus. Although some EVs seem to play an active part in the immune-mediated pathogenesis of musculoskeletal diseases, in contrast, MSC-released EVs might have promising immune therapeutic potential.

One important challenge for EV-mediated immune therapy is the standardization of EV manufacturing. It is commonly accepted that MSCs are a heterogeneous population and that their phenotype differs significantly depending on tissue source and isolation procedure (see section MSC Source and Culture Conditions). Since EVs, at least partly, reflect the immunological molecular composition of the host cells, consistent standardization of the isolation, characterization and expansion of MSCs is of utmost importance in the production of EVs (Thery et al., 2018; Witwer et al., 2019). This aspect will be of particular importance for the near future of EV research as clinical studies will only have little chance of success if reproducibility issues cannot be worked out.

Remaining questions for EV-mediated immune therapy mostly concern the cellular and molecular mechanisms of action and will be the major focus of ongoing research: how do MSC-EVs modulate immune cells and which molecules are involved? As a next step, spatial information about the immune modulatory dynamics of EV products will be needed. This will provide details about the bioavailability as well as cell-specific efficacy of EVs and how both parameters can be optimized to produce more tailored therapeutic interventions with less side effects. Also, novel tools are needed to precisely track EVs *in vivo* to analyze distribution, half-life period and to better forecast effective dosing (Hu et al., 2015b; Vallabhaneni et al., 2015). These details will help to better predict, separate and direct the immune stimulatory vs. anti-inflammatory effects of MSC-EVs, and potentially enable the generation of advanced synthetic EVs for optimized performance (Conlan et al., 2017).

### EV Therapy in Traumatic Joint Injuries and OA

Therapeutic application of MSC-EVs revealed overall positive effects in various situations of musculoskeletal trauma, including repair of osteochondral lesions (Zhang et al., 2016d); *in vivo* studies investigating EV therapies in this context are summarized in Table 1. EV therapy after joint trauma and concurrent cartilage injury, the main risk factor for the development of post-traumatic osteoarthritis (PTOA), is therefore of critical importance to avoid pathogenesis of OA. Up to date, no therapies have been able to halt or delay OA progression satisfactorily or provided effective and long-lasting symptomatic relief. Therapeutic approaches predominantly addressed symptoms and tried to modify/improve structural features of affected joint tissues. Currently, in end stage OA, joint replacement with an artificial prosthesis is the most effective measure to improve pain sensation and quality of life in patients. The development of novel therapeutic approaches targeting the osteoarthritic degradative and inflammatory processes in cartilage, synovium, or bone preferentially at an early stage, requires a deep understanding of the disease status of these joint tissues at the time of the intervention (Grassel and Muschter, 2020).

It has been shown, that MSC-EVs could attenuate OA by stimulation of chondrocyte migration and proliferation (Zhu et al., 2017). In addition, MSC-EVs could protect cartilage and bone from degradation during OA pathogenesis by increasing the expression of chondrocyte markers like collagen II and aggrecan, reducing catabolic markers such as MMP-13 and ADAMTS5, decreasing inflammatory markers (iNOS), protecting chondrocytes from apoptosis, and blocking of macrophage activation (Cosenza et al., 2017). Another important finding of this group was that pre-treatment of MSCs with TGF-β3 can improve the effectiveness of both EVs and microvesicles/microparticles (MPs). Although the differences were relatively small in comparison to non TGF-β3 pre-treated BMSCs, the gene expression pattern was significantly different in the EVs derived from pre-activated BMSCs. Influencing the composition of EV cargo through *ex vivo* pre-treatment and/or pre-activation of MSCs with different factors (e.g., TGF-β, IGF, or FGF) constitute another interesting therapeutic approach to maximize anti-OA potential of EVs. When rat chondrocytes internalized MSC-EVs, this lead to an increase in their proliferation and migration rate that was at least in part mediated via rapid activation of AKT- and ERK signaling through CD73-mediated adenosine signaling (Zhang et al., 2018b).

In accordance with general tissue trauma, traumatic joint injuries induce various pathomechanisms such as oxidative stress, catabolic expression, and synovial inflammation. The inflammatory response is predominantly associated with the acute phase after trauma, and generally attenuates with time, even though some cytokines remain elevated for months or even years (Lieberthal et al., 2015). Likewise, generation and release of other detrimental mediators, comprising pro-inflammatory DAMPs, catabolic proteinases, and ROS, may proceed over years and contribute to chondrocyte death (apoptosis and necroptosis), complement activation, and phenotypical alteration, including senescence and hypertrophy (Jeon et al., 2019; Riegger and Brenner, 2019, 2020; Riegger et al., 2020). Despite the complexity of these processes, recent *in vivo* studies on different animal models of PTOA—critical-size osteochondral defect models, as well as the DMM model—provide the first promising evidence for the therapeutic efficacy of MSC-EVs after traumatic joint injuries (Zhang et al., 2016d, 2018b; Liu et al., 2017b; Chen et al., 2019).

In an osteochondral defect model, combination of hyaluronic acid (HA) and EVs significantly improved hyaline cartilage matrix repair and resulted in mechanically and structurally superior tissue (Wong et al., 2020). Even without HA supplementation, EV treatment of osteochondral defects was found to promote complete restoration of the cartilage and subchondral bone, while preventing formation of fibrous repair tissue (Zhang et al., 2016d). Improved cartilage healing might result from enhanced chondrocyte proliferation and biosynthesis of ECM components (aggrecan, type II collagen), induced by enhanced infiltration of CD163+ regenerative M2 macrophages and subsequent suppression of pro-inflammatory synovial cytokines, as described in a comparable model (Zhang et al., 2018b). Furthermore, it has been observed that even delayed EV administration could reverse DMM-induced post-traumatic cartilage degeneration in mice (Wang et al., 2017; To et al., 2020; Woo et al., 2020). In these studies, EVs derived from human embryonic stem cell-induced MSCs [ESC-MSCs; (Wang et al., 2017)] or small EVs from human adipose-derived stem cells were applied 4 or 5 weeks post-surgery, respectively. Comparable results were found in a combined injury model in rats, including transection of the medial meniscus and the anterior cruciate ligament (Tao et al., 2017). Here, Tao et al. (2017) reported improved efficacy of EVs derived from miR-140-5p-overexpressing synovial MSCs (SMSC) in preventing PTOA development. In contrast, EVs from non-transfected SMSC suppressed the chondrogenic phenotype *in vitro* and exhibited limited chondroprotective effect *in vivo* (Tao et al., 2017). In fact, origin of the donor cells and subsequent EV composition might represent a crucial aspect concerning EV-mediated harm reduction and regeneration after traumatic injury.

Meanwhile, novel EV application techniques have been evolved to improve the healing of osteochondral defects in the context of tissue engineering. As EVs maybe cleared rapidly from joint space, they have been used as therapeutic substances encapsulated and embedded in drug-delivery systems consisting of different matrices, which help to achieve sustained effects. These biomaterials are photo-induced imine crosslinking hydrogel glue, gelatin methacrylate/nanoclay hydrogel or solid 3D printed cartilage ECM/gelatine methacrylate (GelMA) scaffolds (Liu et al., 2017b; Chen et al., 2019; Hu et al., 2020). Liu et al. implanted an acellular tissue patch (EHG) consisting of a hydrogel glue blended with human iPSC-MSC-EVs into a rabbit articular cartilage defect. They observed that EHG retained the EVs and positively influenced chondrocytes and BMSCs *in vitro*. Furthermore, the EHG integrated with the native cartilage matrix and promoted cell deposition at the cartilage defect sites, finally resulting in the promotion of cartilage defect repair. Chen et al. (2019) reported that scaffold-based delivery of EVs enhanced M2 polarization of synovial macrophages, reduced fibrocartilage formation, and effectively restored chondrocyte mitochondrial dysfunction, implying mitoprotective effects (Chen et al., 2019). Mitoprotection can be considered as specific antioxidative potential and has been found to prevent intracellular stress, apoptosis and catabolic processes after cartilage injury (Delco et al., 2018; Bartell et al., 2020).

Overall, stem cell secretomes and EVs applied intra-articularly for the treatment of cartilage pathology in knee OA had pleiotropic and mostly positive effects. Pre-clinical *in vivo* studies in rat, mouse and rabbit OA models resulted in positive effects on the joints and supported the effectiveness of EV intra-articular injections as a minimally invasive therapy. Several miRNAs transported in EVs showed beneficial effects in *in vivo* studies, i.e., miR-92-3p. Mao et al. (2018) investigated the molecular mechanism of exosomal miR-92a-3p and WNT5A in chondrogenesis and cartilage degeneration. They showed that MSC-EV-derived miR-92a-3p inhibited cartilage degradation in their OA mouse model. The authors suggest that miR-92a-3p regulates cartilage development and homeostasis by directly targeting WNT5A. Possibly, EV-derived miR-92a-3p may act as a Wnt inhibitor and exhibits potential as a disease-modifying osteoarthritis drug. Weekly intra-articular injections of EVs from MSCs generated from human ESCs resulted in improved repair of critical-sized osteochondral defects in the femoral groove in immunocompetent rats (Zhang et al., 2018b). Repetitive intra-articular injections of canine BMSC-derived MVs showed promising results in a canine chondral defect model (Sabry et al., 2018). The group reported hat administration of MVs was effective for the functional and morphological recovery of the injured articular cartilage by increased defect filling of the lesion. Accordingly, Ni et al. (2019) demonstrated that intra-articular injection of exosome-like vesicles derived from IL-1β-pretreated primary OA chondrocytes aggravated DMM-induced cartilage erosion and synovial inflammation via miR-449a-5p.

Biodistribution of EVs following intra-articular delivery is not known. Intra-articular administration of neutrophil-derived EVs in a mouse model of inflammatory arthritis showed anti-inflammatory properties and prevented cartilage degradation. Anabolic effects were exerted by penetrating the cartilage ECM to deliver bioactive molecules to the chondrocytes (Headland et al., 2015).

Taken together, intra-articular EV injection might be a promising approach to prevent the development of PTOA and to improve structural damage of joint tissues in chronic OA. Moreover, EVs might enable hyaline cartilage restoration without fibrous tissue formation; thus, facilitating one of the most challenging issues in cartilage regeneration. Considering the poor intrinsic regenerative capacity of adult human articular cartilage, it might be reasonable to apply EVs directly after a traumatic incidence to achieve an early harm reduction and reduce the risk of irreversible cartilage damage and structural tissue alteration.

### EV Therapy in Fracture Healing

Bone regeneration after injury is a complex multiphase process involving many coordinated mechanisms that remove damaged bone and tissue in order to generate a fully integrated structurally competent replacement bone tissue (Hadjidakis and Androulakis, 2006; Raggatt and Partridge, 2010; Loi et al., 2016). There are a number of comorbidities, which affect bone homeostasis and often result in fracture-healing complications, such as ischemia, impaired vascularization, and osteoporosis (Haffner-Luntzer et al., 2019). Complications in bone regeneration are still a major challenge in clinical routine, and thus developing new effective treatments with stronger osteogenic potential and lower incidence of complications is required. Over the last few years, EVs have received significant attention in the field of bone repair and regeneration (Petho et al., 2018). Many pre-clinical studies have revealed the high potential of EV cargo in bone regenerative medicine (see Table 1 for a summary). Furuta et al. (2016) addressed the evaluation of the role of exosomes isolated from human BMSC-conditioned medium in the healing process in a femur fracture model of CD9–/– mice, a strain that is known to produce reduced levels of exosomes. Delayed fracture healing in CD9–/– mice was rescued by the injection of exosomes and was associated with abundant callus formation 2 weeks after fracture. Bone union at 3 weeks after fracture was similar to wild-type mice. In addition, the timing of bone union was significantly shorter in WT mice treated with exosomes compared with the control groups. Tan et al. (2020) published a systematic review of relevant preclinical studies, which were conducted to evaluate the therapeutic efficacy of MSC-EVs for bone regeneration. A total of 23 studies were identified, including a total sample size of 690 rats or mice and 38 rabbits. Generally, it was reported that MSC-EVs were efficacious for bone regeneration in all analyzed animal models of bone defects and diseases and did not show adverse effects. In these studies, MSC-EVs promoted new bone formation with supporting vascularization and displaying improved morphological, biomechanical, and histological outcomes, coupled with positive effects on cell survival, proliferation, migration, osteogenesis, and angiogenesis.

Lately, MSC-EVs combined with various scaffold materials have been shown to generate bone at ectopic sites *in vivo* (Xie et al., 2017) and successfully promoted bone repair in rodent calvarial bone defects (Qi et al., 2016; Qin et al., 2016; Li et al., 2018). Biocompatible scaffolds should degrade at a slow rate and thus facilitate the controlled release of the EVs. Simply loading results in a burst release and will be presumably less efficient as a pro-regenerative approach for bone healing. Qi et al. (2016) reported for a calvarial defect model in osteoporotic rats, that treatment with tricalcium phosphate scaffolds loaded with EVs isolated from human iPSC-MSCs contributed to bone defect repair through induction of angiogenesis and osteogenesis. Li et al. (2018) reported that human AT-MSC-EVs immobilized onto poly-lactic-co-glycolic acid scaffolds exhibited a slow release profile *in vitro* and promoted bone regeneration in a mouse calvarial defect model after 6 weeks *in vivo* (Li et al., 2018).

In conclusion, these studies demonstrate that MSC-EVs and their cargo are therapeutically efficacious in bone regeneration in preclinical animal models and might be considered as next generation therapeutic tools for the treatment of bone pathologies. However, so far only small animal models have been used to study the effects of EVs on bone regeneration and diseases and no large animal studies (i.e., sheep, horses or minipigs) are published. Further studies in large animal models are thus required to establish the safety and efficacy before tackling the design of clinical trials.

## Discussion and Future Perspective

**Figure 4:**
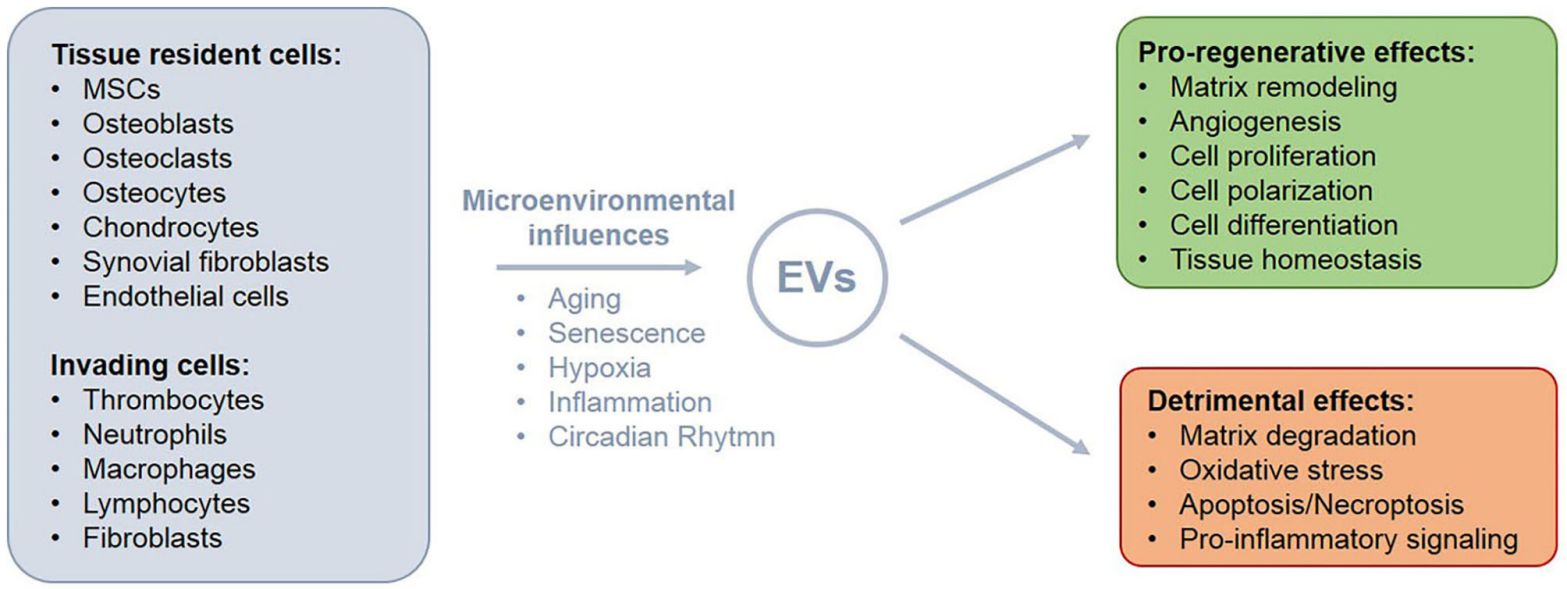
The role of EVs in musculoskeletal diseases—from pro-regenerative to detrimental effects. EVs are an important component of cell communication in musculoskeletal tissues. They can be produced by tissue resident cells as well as by cells, which invade in the process of disease progression and healing. As the parental cells, also EVs and their cargo are affected by microenvironmental changes. EVs can contribute to physiological and pathophysiological processes and thus critically influence tissue homeostasis and regeneration.

EVs are involved in numerous physiological and pathophysiological processes in musculoskeletal tissues. By carrying lipids, proteins and nucleic acids and having the capability to direct them to specific targets (ranging from the extracellular space or the circulation to receptor-mediated cellular uptake), they present an important part of cellular communication within tissues and on a systemic level. It appears that EVs play an important role in tissue homeostasis. Likewise, EVs have been associated with various pathophysiological mechanisms such as matrix degradation or pro-inflammatory effects in musculoskeletal disorders (Figure 4) and as such, have been proposed as biomarkers. On the other hand, EVs contribute to tissue regeneration by mitogenic, angiogenic and immune modulatory effects, which renders them as potential therapeutics in regenerative medicine (an overview of studies investigating EV-based therapies for musculoskeletal disorders is provided in Table 1). In common for all those features of EVs is that we still lack the full insight in underlying mechanisms and functional active components. Also, it is not fully understood how the parental cell produces EVs and incorporates the potential therapeutic effective molecules into the EVs. A detailed understanding of how the recipient cell internalizes EVs is critical to increase therapeutic effects of EVs and to develop highly efficient EVs for drug delivery and even gene therapy. This will be a requirement for the translation of EV-based procedures to clinical application.

The biggest hurdle in EV research so far are inconsistent preparation and characterization methods, which have been addressed by the ISEV by suggesting specific experimental guidelines (Lotvall et al., 2014; Thery et al., 2018). While still not implemented in all studies, these guidelines will help to clearly assign functional outcomes to EVs. Other practical considerations have to be addressed, such as potential contamination of EV preparations with particles contained in FBS or platelet products, or guaranteeing maintenance of viability and functions of parental cells when cultured with depleted serum preparations or even under serum-free conditions. Finally, very little is currently known about the therapeutic efficient dose of EVs, which will have to be determined separately for various applications and might lead to challenges when the EV production process has to be dramatically scaled-up.

Currently, mostly small animal models have been used to study the effects of EVs on musculoskeletal diseases and no large animal studies are published yet. Consequently, the next step should be the involvement of large animal models to establish the safety and efficacy of the different musculoskeletal EV therapy approaches. Large animal studies as in sheep, horses or minipigs are commonly the prerequisite for designing first clinical trials evaluating the therapeutic benefits of EVs in human musculoskeletal disorders.

Nevertheless, there is a big need for new therapeutic strategies in musculoskeletal diseases as incidences are increasing with an ever-growing aging population and cell-based therapies have shown limited success so far. In this light, EV-based therapies, which can circumvent many of the disadvantages related to cell therapies have a tremendous potential. EV-based therapies may also benefit from EV-engineering approaches that aim at modulating either the cargo or the targeting of EVs in order to improve their therapeutic efficiency. This includes the use of EVs as drug delivery vehicle and particularly for the delivery of small molecules or nucleic acids as reviewed elsewhere (Ramasubramanian et al., 2019). Incorporation in EVs can overcome also problems arising from low solubility or bioavailability of molecules, as demonstrated for the anti-inflammatory agent curcumin (Sun et al., 2010; Moballegh Nasery et al., 2020). Moreover, it has been proposed that pre-conditioning (e.g., by hypoxia or inflammatory factors) of parental cells may be applied to modulate the EV cargo and in turn their therapeutic success with regards of e.g., angiogenic or immunomodulatory effects (Xue et al., 2018; Martin-Rufino et al., 2019). Such strategies have a great potential for the development of efficient therapies for musculoskeletal diseases but yet need further research efforts into the underlying mechanisms as discussed above.

## Author Contributions

All authors have contributed to writting and editing of the manuscript and have approved the final version.

## Conflict of Interest

The authors declare that the research was conducted in the absence of any commercial or financial relationships that could be construed as a potential conflict of interest.
